# GLP-1 receptor signaling increases PCSK1 and **β** cell features in human **α** cells

**DOI:** 10.1172/jci.insight.141851

**Published:** 2021-02-08

**Authors:** Mridusmita Saikia, Marlena M. Holter, Leanne R. Donahue, Isaac S. Lee, Qiaonan C. Zheng, Journey L. Wise, Jenna E. Todero, Daryl J. Phuong, Darline Garibay, Reilly Coch, Kyle W. Sloop, Adolfo Garcia-Ocana, Charles G. Danko, Bethany P. Cummings

**Affiliations:** 1 Department of Biomedical Sciences and; 2 Baker Institute for Animal Health, Cornell University College of Veterinary Medicine, Ithaca, New York, USA.; 3 Cayuga Medical Center, Ithaca, New York, USA.; 4 Diabetes and Complications, Lilly Research Laboratories, Lilly, Indianapolis, Indiana, USA.; 5 Icahn School of Medicine at Mount Sinai, New York, New York, USA.

**Keywords:** Endocrinology, Metabolism, Diabetes, Islet cells

## Abstract

Glucagon-like peptide-1 (GLP-1) is an incretin hormone that potentiates glucose-stimulated insulin secretion. GLP-1 is classically produced by gut L cells; however, under certain circumstances α cells can express the prohormone convertase required for proglucagon processing to GLP-1, prohormone convertase 1/3 (PC1/3), and can produce GLP-1. However, the mechanisms through which this occurs are poorly defined. Understanding the mechanisms by which α cell PC1/3 expression can be activated may reveal new targets for diabetes treatment. Here, we demonstrate that the GLP-1 receptor (GLP-1R) agonist, liraglutide, increased α cell GLP-1 expression in a β cell GLP-1R–dependent manner. We demonstrate that this effect of liraglutide was translationally relevant in human islets through application of a new scRNA-seq technology, DART-Seq. We found that the effect of liraglutide to increase α cell PC1/3 mRNA expression occurred in a subcluster of α cells and was associated with increased expression of other β cell–like genes, which we confirmed by IHC. Finally, we found that the effect of liraglutide to increase bihormonal insulin^+^ glucagon^+^ cells was mediated by the β cell GLP-1R in mice. Together, our data validate a high-sensitivity method for scRNA-seq in human islets and identify a potentially novel GLP-1–mediated pathway regulating human α cell function.

## Graphical abstract

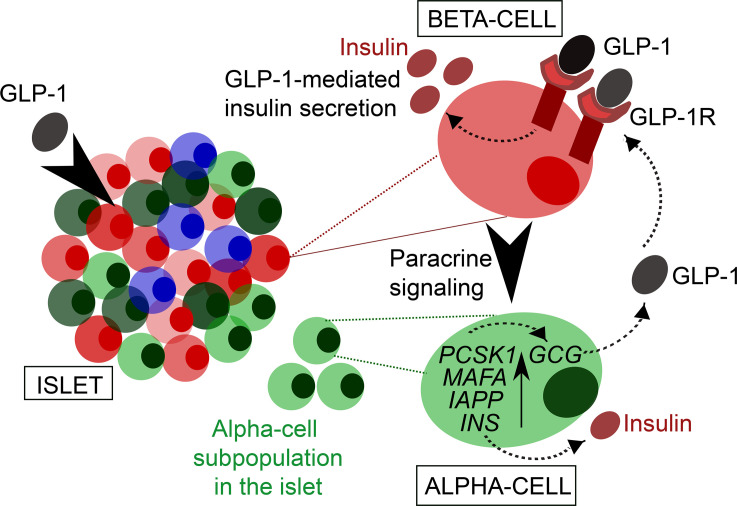

## Introduction

Islet dysfunction is the defining step in the development of type 2 diabetes mellitus (T2DM); it consists of impaired β cell insulin production and increased α cell glucagon production (1–3). The proglucagon-derived peptides, glucagon and glucagon-like peptide-1 (GLP-1), are key regulators of glucose-stimulated insulin secretion (GSIS) (4–9). However, GLP-1, in addition to various GLP-1R agonists, is a 300-fold stronger inducer of GSIS (4, 9) and does not produce the effects of glucagon to increase hepatic glucose production, which is thought to be a key pathogenic driver of T2DM (3). Proglucagon is expressed in gut L cells and islet α cells and is differentially cleaved. Prohormone convertase 1/3 (protein, PC1/3; gene, *PCSK1*) is expressed in L cells to produce GLP-1, while prohormone convertase 2 (protein, PC2; gene, *PCSK2*) is expressed in α cells to produce glucagon. Under certain conditions, α cells can express *PCSK1* and produce active GLP-1, but the mechanisms regulating α cell *PCSK1* expression are poorly defined.

This knowledge gap is particularly interesting in light of an increasing body of literature suggesting that the mechanisms by which GLP-1 exerts its incretin effect are more complex than previously thought. In the classical model, L cell–derived GLP-1 is secreted in response to feeding and acts as an endocrine hormone to signal through the β cell GLP-1R to potentiate GSIS (10). However, this model has several limitations. For example, circulating GLP-1 concentrations are low and postprandial increases are minimal (11). Furthermore, active GLP-1 is rapidly degraded by dipeptidyl peptidase IV (DPP-IV) (12–15). Together, these observations point to a mismatch between circulating GLP-1 levels and GLP-1 function.

Under normoglycemic conditions, adult α cells express low levels of PC1/3 and produce little GLP-1 (16). However, studies suggest that α cell–derived GLP-1 is an important metabolic regulator. For example, reexpression of *Gcg* in whole-body *Gcg*-KO mice in the pancreas, but not the gut, results in differential metabolic phenotypes (17–19). Furthermore, work in an α cell–specific PC1/3–KO mouse model demonstrates that loss of α cell–derived GLP-1 impairs the response to metabolic stress (20). Moreover, β cell destruction by streptozotocin is ameliorated by PC1/3 upregulation in α cells in mice, suggesting that locally produced GLP-1 may contribute to β cell adaptation (21). Finally, genetic modification of α cells to increase PC1/3 expression has been shown to improve glucose regulation in animal models (21, 22). These results strongly suggest a role for α cell–derived GLP-1 in metabolic regulation.

Herein, we report that pharmacologic activation of the β cell GLP-1R induced α cell PC1/3 and GLP-1 expression in mice. We demonstrate that this effect of a GLP-1R agonist was translationally relevant in human islets through application of a single-cell RNA-Seq (scRNA-Seq) technology, droplet-assisted RNA targeting by single-cell sequencing (DART-Seq) (23), a high-sensitivity scRNA-Seq approach. scRNA-Seq platforms often suffer from limitations in achieving adequate depth of sequencing to accurately measure lowly expressed transcripts. We were particularly concerned about this limitation, as *PCSK1* is lowly expressed in adult α cells (16). Here, we show that DART-Seq overcomes this limitation; we found that the effect of a GLP-1R agonist to increase α cell *PCSK1* mRNA expression occurred in a subcluster of α cells and was associated with increased expression of markers of β cell fate. Finally, we found that the effect of a GLP-1R agonist to increase bihormonal insulin^+^ glucagon^+^ cells was mediated by the β cell GLP-1R in mice. Together, our data validate a highly sensitive scRNA-Seq method in human islets and identify a potentially novel pathway regulating human α cell function.

## Results

### β Cell GLP-1R signaling increases α cell PC1/3 and GLP-1 expression.

**Figure 1:**
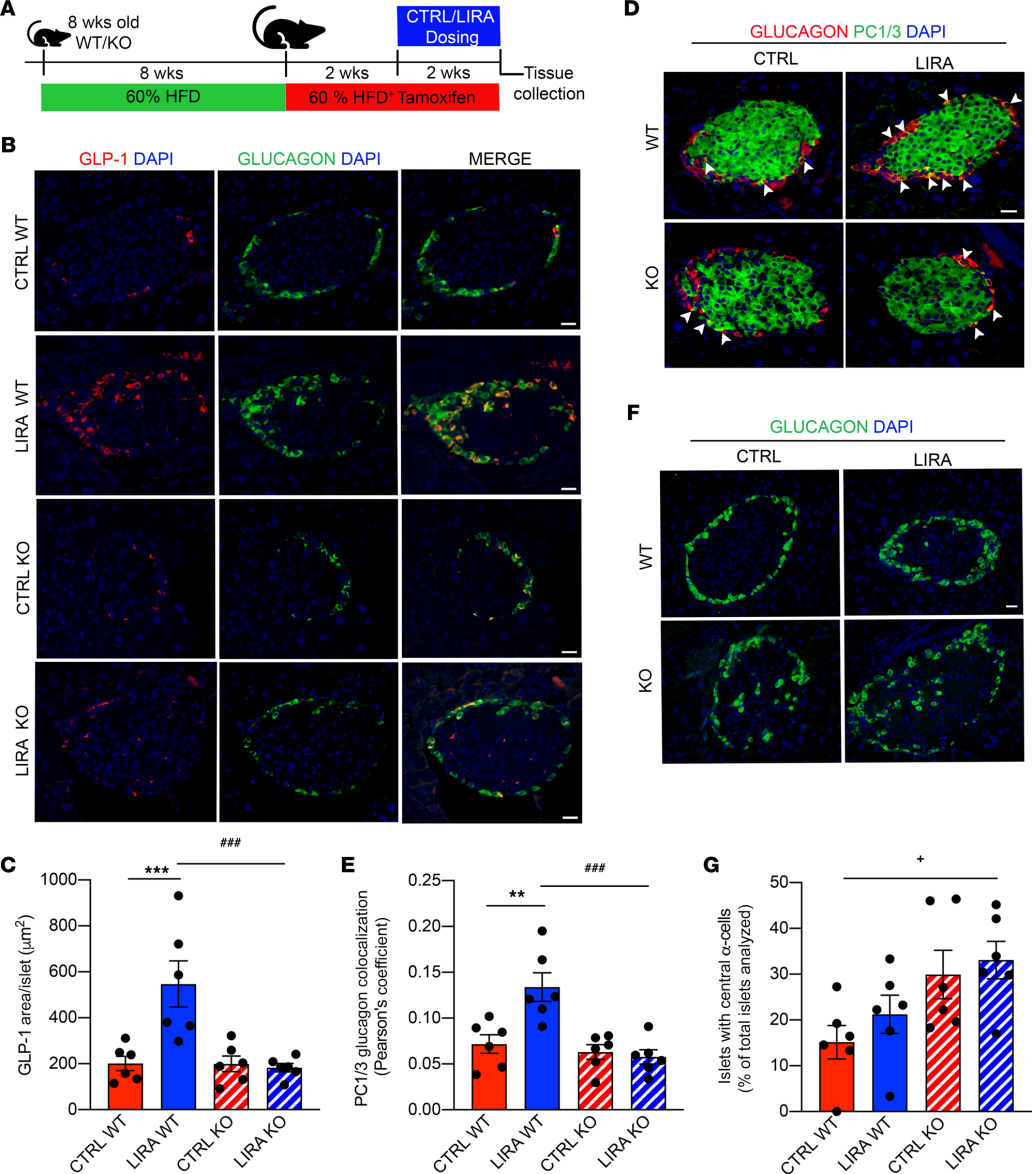
β Cell GLP-1R signaling increases islet GLP-1 production and PC1/3 expression. (**A**) Schematic of the mouse study design. (**B**) Representative images of mouse pancreas sections immunostained for GLP-1 (red), glucagon (green), and DAPI in WT and KO mice treated with saline (CTRL) or liraglutide (LIRA). (**C**) Average GLP-1 staining per islet. (**D**) Representative images of pancreas sections immunostained for glucagon (red), PC1/3 (green), and DAPI. Examples of glucagon and PC1/3 colocalization are indicated by white arrows. (**E**) PC1/3 colocalization with glucagon. (**F**) Representative images of pancreas sections immunostained for glucagon (green) and DAPI. (**G**) Percentage of islets with centrally located α cells. Data are presented as mean ± SEM. *n* = 6 per group. **P < 0.01, ***P < 0.001 CTRL WT vs. LIRA WT, ^###^*P* < 0.001 LIRA WT vs. LIRA KO by 2-factor ANOVA. ^+^*P* < 0.05 CTRL WT vs. CTRL KO by 2-tailed Student’s *t* test. Scale bar: 20 μm. See also Supplemental Figures 1 and 6.

We previously found that bariatric surgery increases α cell GLP-1 and PC1/3 expression in a β cell GLP-1R–dependent manner (24). Whether this effect of bariatric surgery can be induced by pharmacologic stimulation of the β cell GLP-1R remains unknown. Therefore, we evaluated the effect of the GLP-1R agonist, liraglutide, on α cell GLP-1 production and PC1/3 expression using a tamoxifen-inducible β cell–specific GLP-1R–KO mouse model (9, 25). Starting at 8 weeks of age, male and female β cell GLP-1R WT (*Glp-1r*^β^ ^cell+/+^ [WT]) and KO (*Glp-1r*^β^ ^cell–/–^** [KO]) mice were fed a high-fat diet (HFD) for 6 weeks to produce an obese insulin-resistant phenotype (Figure 1A). Following 6 weeks of HFD, all mice were switched to HFD supplemented with tamoxifen to induce β cell GLP-1R knockdown and were maintained on this diet throughout study, resulting in a total duration of 8 weeks of HFD feeding before intervention, which results in impaired glucose tolerance, insulin secretion, and islet function, as previously established (26–28). Two weeks after the start of the tamoxifen HFD diet, mice were given twice daily injections (200 μg/kg of body weight subcutaneous) of either liraglutide or saline for 2 weeks. At the end of the study, mice were fasted overnight (12 hours) and euthanized for tissue collection. While liraglutide decreased cumulative food intake, body weight and white adipose depot weights did not differ between treatment or genotype, likely due to the short period of liraglutide treatment (Supplemental Figure 1, A–D; supplemental material available online with this article; https://doi.org/10.1172/jci.insight.141851DS1). Brown adipose depot weight was elevated by liraglutide treatment in WT mice but not KO mice (Supplemental Figure 1E; *P* < 0.05). Additionally, liraglutide treatment lowered fasting blood glucose levels in WT mice but not in KO mice (Supplemental Figure 1F; *P* < 0.05).

To assess the effect of liraglutide treatment and the role of β cell GLP-1R signaling on α cell GLP-1 expression, pancreatic sections from these mice were costained for GLP-1 and glucagon, using an antibody that has been rigorously validated to be specific for GLP-1 (29). Liraglutide treatment increased islet GLP-1 staining compared with saline in WT mice but not in KO mice (Figure 1, B and C; *P* < 0.01). Furthermore, islet GLP-1 staining was higher in liraglutide​-treated WT mice compared with that in liraglutide-​treated KO mice (Figure 1, B and C; *P* < 0.01). Based on these results, we assessed α cell PC1/3 expression by costaining pancreas sections for PC1/3 and glucagon. Consistent with the GLP-1 data, liraglutide treatment increased PC1/3 colocalization with glucagon compared with that in saline-treated controls in WT mice but not in KO mice (Figure 1, D and E; *P* < 0.01). Furthermore, PC1/3 colocalization with glucagon was elevated in liraglutide​-treated WT mice compared with that in liraglutide-​treated KO mice (Figure 1, D and E; *P* < 0.01). We have previously found that β cell GLP-1R–KO leads to an increased number of islets containing centrally located α cells, suggesting that the β cell GLP-1R regulates normal islet morphology (24). Consistent with this, the number of islets containing centrally located α cells was proportionally increased in β cell GLP-1R–KO mice compared with that in WT mice (Figure 1, F and G; *P* < 0.05). Liraglutide treatment did not effect α cell location relative to that in control groups (Figure 1, F and G). These findings highlight the potential importance of β cell GLP-1R signaling for the maintenance of normal islet morphology. Together, our data show that liraglutide treatment increases α cell GLP-1 and PC1/3 expression in a β cell GLP-1R–dependent manner in mice.

### DART-Seq improves detection of low-abundance transcripts in human islets.

A growing body of literature reports that turning on α cell GLP-1 production potently improves islet function and glucose homeostasis (10, 16, 17, 24, 30, 31). PC1/3 expression is required for α cell GLP-1 production; however, the mechanisms regulating α cell PC1/3 expression are poorly defined. Therefore, we sought to determine the translational relevance of our data showing that β cell GLP-1R signaling increases α cell GLP-1 production and PC1/3 expression in mice.

**Figure 2:**
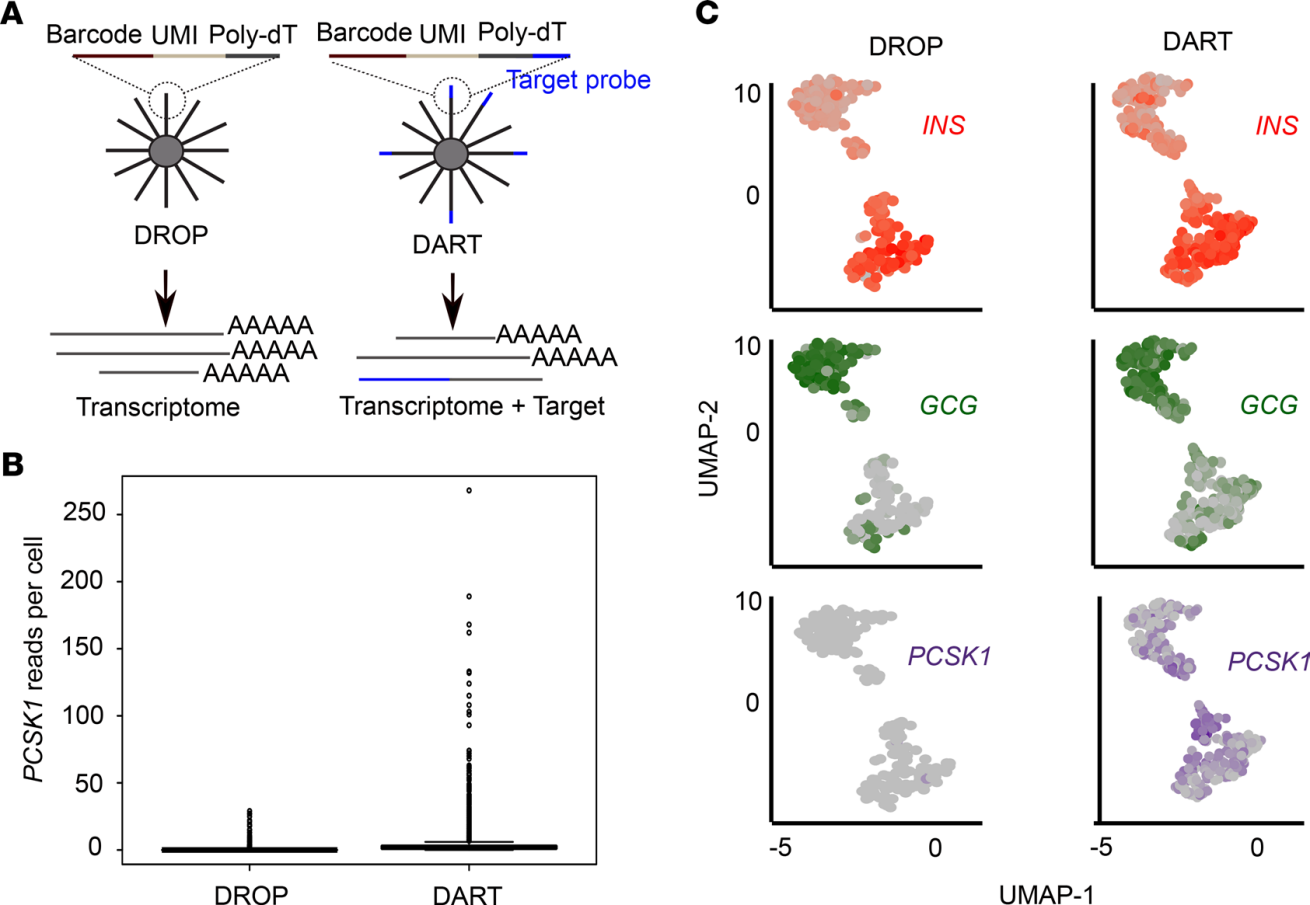
DART-Seq improves detection of low-abundance transcripts in human islets. (**A**) Schematic of DART-Seq modification of the DROP-Seq nano bead. (**B**) Box plot showing *PCSK1* reads per cell detected by DROP-Seq and DART-Seq. (**C**) Expression level of *INS* (red), *GCG* (green), and *PCSK1* (purple) projected on the UMAP generated from human islet cells using DROP-Seq and DART-Seq, respectively. *n* = 1 per group. See also Supplemental Figure 2 and Supplemental Table 1.

To take β cell and α cell heterogeneity (32, 33) into account in our analysis, we used a scRNA-Seq platform. However, scRNA-Seq platforms often suffer from limitations in achieving adequate depth of sequencing to accurately measure lowly expressed transcripts (34). This was of particular concern for our studies, as our primary aim was to assess α cell *PCSK1* expression, which is a lowly expressed transcript in α cells. To address this, we used a recently described scRNA-Seq platform, DART-Seq (23). DART-Seq is a modified version of DROP-Seq (35), which improves sensitivity by simultaneously measuring the transcriptome as well as targeting specific RNA molecules by using beads that carry a mix of poly-deoxythymidine (poly-dT) and specific probes in their tails (Figure 2A). To assess the efficiency of *PCSK1* detection using DART-Seq, we generated DART-Seq beads with poly-dT and *PCSK1* probes. As additional controls, we added proglucagon (*GCG*) and insulin (*INS*) probes on these beads (probe sequences in Supplemental Table 1).

To validate the application of DART-Seq to human islets, islets from a healthy donor were equally divided into 2 samples and each sample was subjected to either DROP-Seq or DART-Seq (Supplemental Figure 2). Compared with DROP-Seq, DART-Seq produced a higher median signal value as well as a higher signal in individual cells (Figure 2B). Similarly, unsupervised clustering demonstrated that DART-Seq was more effective at detecting *PCSK1* transcripts compared with DROP-Seq (Figure 2C). With DART-Seq, *PCSK1* was detected predominantly in β cells but also in a few α cells (Figure 2C). This is consistent with previous reports assessing PC1/3 expression in human islets (30). DART-Seq also detected a higher number of β and α cells compared with DROP-Seq (Figure 2C). This improvement in single-cell detection was likely the result of the *INS* and *GCG* probes included in our DART-Seq beads. Together, these data demonstrate that DART-Seq enhances the detection of low-abundance transcripts in human islets. Therefore, we used DART-Seq to examine the effect of liraglutide on α cell *PCSK1* expression in human islets.

### A GLP-1R agonist does not effect the relative abundance of endocrine cell types in human islets.

DART-Seq was performed on islets obtained from 3 healthy male donors (Supplemental Figure 3A). Islets from each donor were equally divided into 2 samples, and each sample was treated with either saline or liraglutide for 12 hours. Islets were then dissociated into a single-cell suspension and subjected to DART-Seq using the bead design validated in Figure 2. We combined the single-cell transcriptome data obtained from all 3 donors and performed unsupervised clustering on the combined data set using Seurat (36) to create various cell clusters and then used respective hormone markers to identify each cluster (Supplemental Figure 3, B and C). To combine cell-type clustering with the assessment of how liraglutide treatment affected cell type–specific changes in gene expression, the Seurat integration strategy was used (37). This integrated analysis allowed for the identification of the islet cell types while performing comparative analyses between control and liraglutide conditions.

**Figure 3:**
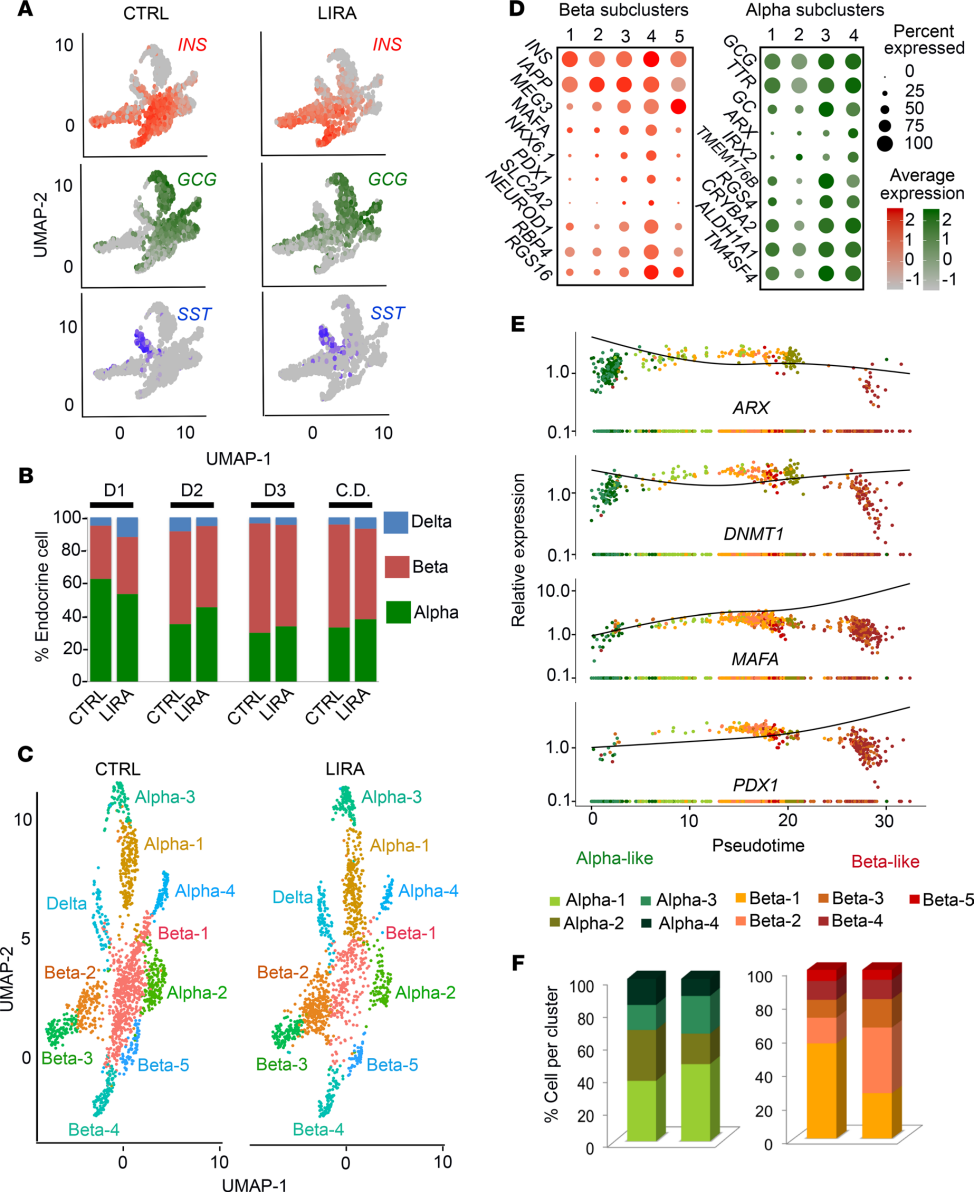
DART-Seq assessment of human islets treated with saline or a GLP-1R agonist. (**A**) Expression level of *INS* (red), *GCG* (green), and *SST* (blue) projected on the UMAP generated from human islets treated with saline (CTRL) or liraglutide (LIRA). (**B**) Percentage of α, β, and δ cells in saline- and liraglutide-treated islets from the 3 individual donors (D1–D3) and in the combined data set (CD). (**C**) UMAP projection of all endocrine cells with respective subclusters in saline- and liraglutide-treated islets. (**D**) Dot plots showing the expression level of key identity genes in α cell (right) and β cell (left) subclusters. (**E**) Pseudotime trajectory of α and β cell subclusters showing relative expression of key identity genes, *ARX*, *DNMT1*, *MAFA*, and *PDX1*. (**F**) Percentage of α and β cell subclusters in saline- and liraglutide-treated islets. Percentage calculated using combined data set. *n* = 3 per group. See also Supplemental Figure 3 and Supplemental Table 2.

Clustering resulted in a clear separation of the endocrine and the exocrine cells (Supplemental Figure 3, B and C). We focused on the changes within the endocrine population of the islet (Figure 3, A and B) and determined the proportion of α, β, and δ cells relative to total endocrine cells in each donor. Due to the low expression of pancreatic polypeptide (*PPY*) and ghrelin (*GHR*), the γ and ε cells did not form distinct clusters, thereby preventing us from quantifying their proportions. The proportion of α and β cells varied from donor to donor (Figure 3B), which is consistent with previous scRNA-Seq studies (33, 38). Additionally, the combined data showed no difference in the relative proportions of α, β, and δ cells between treatment (Figure 3B).

### Genes related to cell identity and maturity drive α and β cell heterogeneity.

In accordance with recent scRNA-Seq studies (32, 33, 38, 39), our DART-Seq data revealed that α and β cells exhibit substantial heterogeneity (Figure 3C). To determine which genes drive the differences in transcriptional landscape among the various subclusters, we focused on functionally related genes that exhibited high differential expression between each subcluster within individual endocrine cell populations as well as other known β and α cell marker genes.

Functional heterogeneity among β cells can occur with regard to glucose threshold and insulin secretory response of individual β cells (40–42). Consistent with this, expression levels of the long noncoding RNA, maternally expressed 3 (*MEG3*) and glucose transporter 2 (*SLC2A2*), both of which contribute to insulin synthesis and secretion (43–46), increased in a step-wise fashion from the β-1 to β-4 subclusters (Figure 3D). Similar to other scRNA-Seq studies of islets (32), expression levels of retinol binding protein 4 (*RBP4*) were associated with β cell subcluster heterogeneity, with the highest expression levels in β-4 and the lowest expression levels in β-2 (Figure 3D).

The primary functionally related group of genes differentially expressed across subclusters was related to β cell identity and maturity. For example, *INS*, islet amyloid polypeptide (*IAPP*), MAF bZIP transcription factor A (*MAFA*), pancreatic and duodenal homeobox 1 (*PDX1*), and NK6 homeobox 1 (*NKX6.1*), exhibited a varying degree of expression in β cell subclusters, with the highest expression in β-4 cells (Figure 3D). Moreover, expression levels of neuronal differentiation 1 (*NEUROD1*), which is a transcription factor with a critical role in β cell fate and maturation (47), follow similar patterns to the aforementioned genes, with the highest expression in the β-4 subcluster (Figure 3D). Expression levels of regulator of G protein signaling 16 (*RGS16*) increased in a step-wise fashion from the β-1 to β-4 subclusters (Figure 3D). *RGS16* is a regulator of G protein signaling that is expressed in pancreatic progenitor cells and endocrine cells during development but is then silenced during adulthood; however, studies have shown that exendin-4, a GLP-1R agonist, promotes reexpression of *RGS16* in β cells to promote proliferation (48).

Similar to the β cell subclusters, a key functionally related group of genes differentially expressed across the α cell subclusters were related to α cell identity and maturity. α Cell subclusters exhibited a varying degree of *GCG* expression, with the highest expression observed in the α-3 and α-4 subclusters (Figure 3D). Similarly, expression of α cell signature genes, transthyretin (*TTR*), vitamin D–binding protein (*GC*), crystallin β A2 (*CRYBA2*), aldehyde dehydrogenase 1 family member A1 (*ALDH1A1*), and transmembrane 4 L 6 family member 4 (*TM4SF4*), was highest in the α-3 and α 4 subclusters (Figure 3D). A similar expression pattern was exhibited for the regulator of *RGS4*, a regulator of G protein signaling that functions as a negative regulator of insulin release from β cells (49) but has been previously associated with α cell heterogeneity (32). Furthermore, transcripts that regulate gene expression to promote α cell maturity, such as aristaless related homeobox (*ARX*) and Iroquois homeobox 2 (*IRX2*) (50–52), were increased in a step-wise fashion across α-1 to α-4 cells (Figure 3D). Together, these data demonstrate that differences in gene expression primarily related to cell identity and maturity are key drivers of heterogeneity within α cell and β cell subpopulations.

To ascertain whether the heterogeneity observed within the α and β cells is an indication of cellular processes, such as differentiation, we performed pseudotime analysis to determine the pseudotemporal order within these subclusters (53). We plotted the continuous change in expression of α and β cell identity markers *ARX,* DNA methyltransferase 1 *(DNMT1*), *MAFA*, and *PDX1* in the α and β cell subclusters (Figure 3E). The α-4 and α-3 subclusters were at one end of the pseudotime trajectory (Figure 3E). These subclusters exhibited the highest expression of *ARX* and *DNMT1*, suggesting that these subpopulations represent the most mature α cell subclusters (Figure 3E; *P* < 0.001). We denote this end of the trajectory as the most “α like.” The β-3, β-4, and β-5 subclusters were at the other end of the pseudotime trajectory, exhibiting the highest expression of *MAFA* and *PDX1*, suggesting that these subpopulations were the most mature β cell subpopulations. We denote this end of the trajectory as the most “β like.” Despite higher expression of *GCG* than *INS*, the α-2 subcluster was located closer to the “β-like” end of the trajectory. One potential explanation for this finding is the high levels of stress-induced alterations in gene expression (e.g., increased expression of mitochondrial transcripts) within this subcluster. Indeed, a previous report shows that cellular stress can promote non–β cells in the islet to exhibit β cell–like properties (54). The remaining subclusters, α-1, β-1, and β-2, were located in the middle of the trajectory (Figure 3E), suggesting that these subclusters maintain a more flexible cell identity.

To determine whether liraglutide treatment changes the proportions of individual α and β cell subclusters, we used the number of cells in each subcluster to calculate their respective percentages. Liraglutide treatment increased the percentage of the α-1 and α-3 subclusters (Figure 3F). This increase was accompanied by a decrease in the α-2 and α-4 subclusters. Within the β cell population, liraglutide treatment increased the β-2 and β-3 subclusters and decreased the β-1 subcluster. The relative proportions of the β-4 and β-5 subclusters remained unaltered (Figure 3F). Based on the liraglutide-induced expansion of the α-1 subcluster, we further focused on this subcluster.

### A GLP-1R agonist increases PCSK1 expression and markers of β cell fate in a subcluster of α cells.

**Figure 4:**
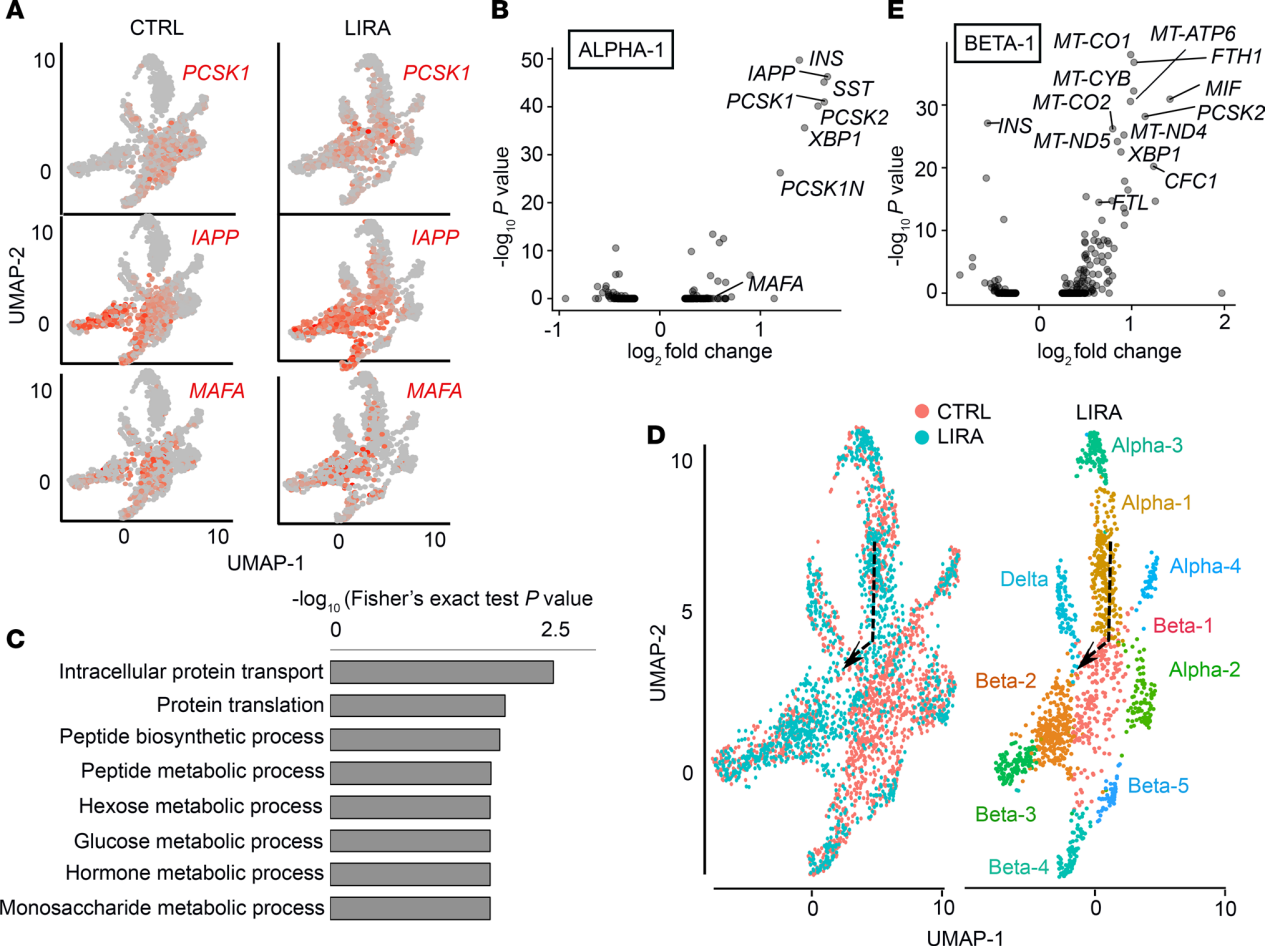
A GLP-1R agonist increases *PCSK1* expression and markers of β cell fate in a subcluster of α cells. (**A**) Expression levels of *PCSK1*, *IAPP*, and *MAFA* projected on the UMAP generated from human islet cells treated with saline (CTRL) or liraglutide (LIRA). (**B**) Volcano plot showing genes that are differentially regulated in the α-1 subcluster upon liraglutide treatment. (**C**) GO term analysis showing the biological processes enriched in the α-1 subcluster. (**D**) Different representation of data presented in Figure 3C. Left: Saline- (CTRL, red) and liraglutide-treated (blue) islet cells projected on the UMAP and colored by treatment. Right: Subclusters of α and β cells in the liraglutide sample with the arrow indicating proposed direction of change. (**E**) Volcano plot showing genes that are differentially regulated in the β-1 subcluster upon liraglutide treatment. *n* = 3 per group. See also Supplemental Figure 4.

Consistent with our findings in mice, liraglutide treatment caused a 1.6 log–fold increase in *PCSK1* expression in the α-1 subcluster compared with control (Figure 4, A and B, and Supplemental Figure 4A; *P* < 0.001). Further investigation of differential gene expression in α-1 cells revealed that the most highly upregulated transcripts in response to liraglutide treatment were *INS* and *IAPP* (Figure 4, A and B, and Supplemental Figure 4A; *P <* 0.001). Liraglutide treatment also increased the expression of the β cell transcription factor, *MAFA*, in the α-1 subcluster compared with control (Figure 4, A and B, and Supplemental Figure 4A; *P <* 0.001). The other 3 α cell subclusters exhibited either a nonsignificant change (α-3) or a significant decrease (α-2, α-4) in *INS* expression upon liraglutide treatment (Supplemental Figure 4A). Therefore, the α-1 subcluster was the only α cell subcluster to exhibit a consistent increase in *IAPP*, *PCSK1*, and *MAFA* in response to liraglutide treatment, suggesting that the effect of liraglutide to increase expression of these β cell genes was specific to the α-1 subcluster. Another notable transcript upregulated in the α-1 subcluster in response to liraglutide treatment was somatostatin (*SST*) (Figure 4B). Coexpression of insulin and somatostatin has been observed in reprogrammed pancreatic organoid cells in conditions promoting differentiation (55). Although we observed liraglutide-induced overexpression of *SST* in the α-1 subcluster compared with control, gene expression in δ cells was largely unaffected by liraglutide treatment (Supplemental Figure 4B). We used gene ontology (GO) term analysis to determine the biological processes induced by liraglutide treatment in the α-1 subcluster. Liraglutide treatment enriched GO terms related to intracellular protein transport and several metabolic pathways (Figure 4C).

Liraglutide treatment protects islets against endoplasmic reticulum (ER) stress (56). To determine whether liraglutide treatment affects ER stress in specific islet cell populations, we included probes for 3 ER stress genes in our DART-Seq bead design (Supplemental Table 1). These 3 genes, X box binding protein 1 (*XBP1*), activating transcription factor 4 (*ATF4*), and *ATF6*, are regulators of the 3 pathways of the unfolded protein response (UPR) (57, 58). Liraglutide treatment increased total *XBP1* expression in the α-1, α-3, and α-4 subclusters compared with control (*P* < 0.001). Liraglutide treatment did not alter *XBP1* expression in α-2 cells. Since the α-2 subcluster is composed of cells exhibiting transcriptional signs of stress and the proportion of this subcluster was decreased upon liraglutide treatment, these data suggest that liraglutide-induced expression of *XBP1* in the α-1, α-3, and α-4 subclusters is a reflection of its protective action. Liraglutide treatment did not alter α cell expression of *ATF4* or *ATF6* compared with saline.

Uniform manifold approximation and projection (UMAP) visualization showed that liraglutide treatment shifted the α-1 subcluster toward the β-1 subcluster compared with control (Figure 4D, dotted arrow). Therefore, we determined which genes were differentially regulated in the β-1 subcluster (Figure 4E). The β-1 subcluster exhibited increased expression of several mitochondrial genes as well as stress-related genes, such as *XBP1*, ferritin heavy chain 1 (*FTH1*), and ferritin light chain (*FTL*), in response to liraglutide treatment. *INS* expression was significantly reduced in these cells (Figure 4E; *P* < 0.001). Recent studies have shown that a high insulin demand causes some β cells to go into a high-stress, low-insulin secretion phase (59). Our data suggest that the β-1 subcluster represents that class of β cells. Liraglutide treatment reduced the proportion of the β-1 subcluster, further highlighting the protective role of liraglutide (Figure 3F).

Our current and previous findings in mice suggest that β cell GLP-1R signaling promotes the secretion of a factor that can act on α cells to turn on α cell *PCSK1* expression (24). Therefore, we examined the genes with the greatest log-fold increase in response to liraglutide treatment in β cells, which encode a secreted factor. Using this criterion, we found that *MIF* (encoding macrophage migration inhibitory factor, 1.4 log–fold increase in β-1), *SERPINA1* (encoding α-1-antitrypsin, 0.8 log–fold increase in β-1, 0.4 log–fold increase in β-2), *REG1A* (encoding lithostathine-1-α, 0.6 log–fold increase in β-2), and *GLS* (encoding glutaminase, 0.7 log–fold increase in β-2) are the genes encoding a secreted protein with the greatest increase in β cells in response to liraglutide treatment (Supplemental Table 2). β Cells secrete *MIF*, which regulates insulin secretion in an autocrine fashion (60). *SERPINA1* deficiency has been associated with increased risk for T2DM in humans and treatment with *SERPINA1* protects β cell mass in mice (61–63). *REG1A* is associated with β cell regeneration (64). *GLS* hydrolyses glutamine to glutamate, which is a key regulator of α cell secretion (65) and an immediate precursor to γ-aminobutyric acid (GABA) secreted from β cells (66).

**Figure 5:**
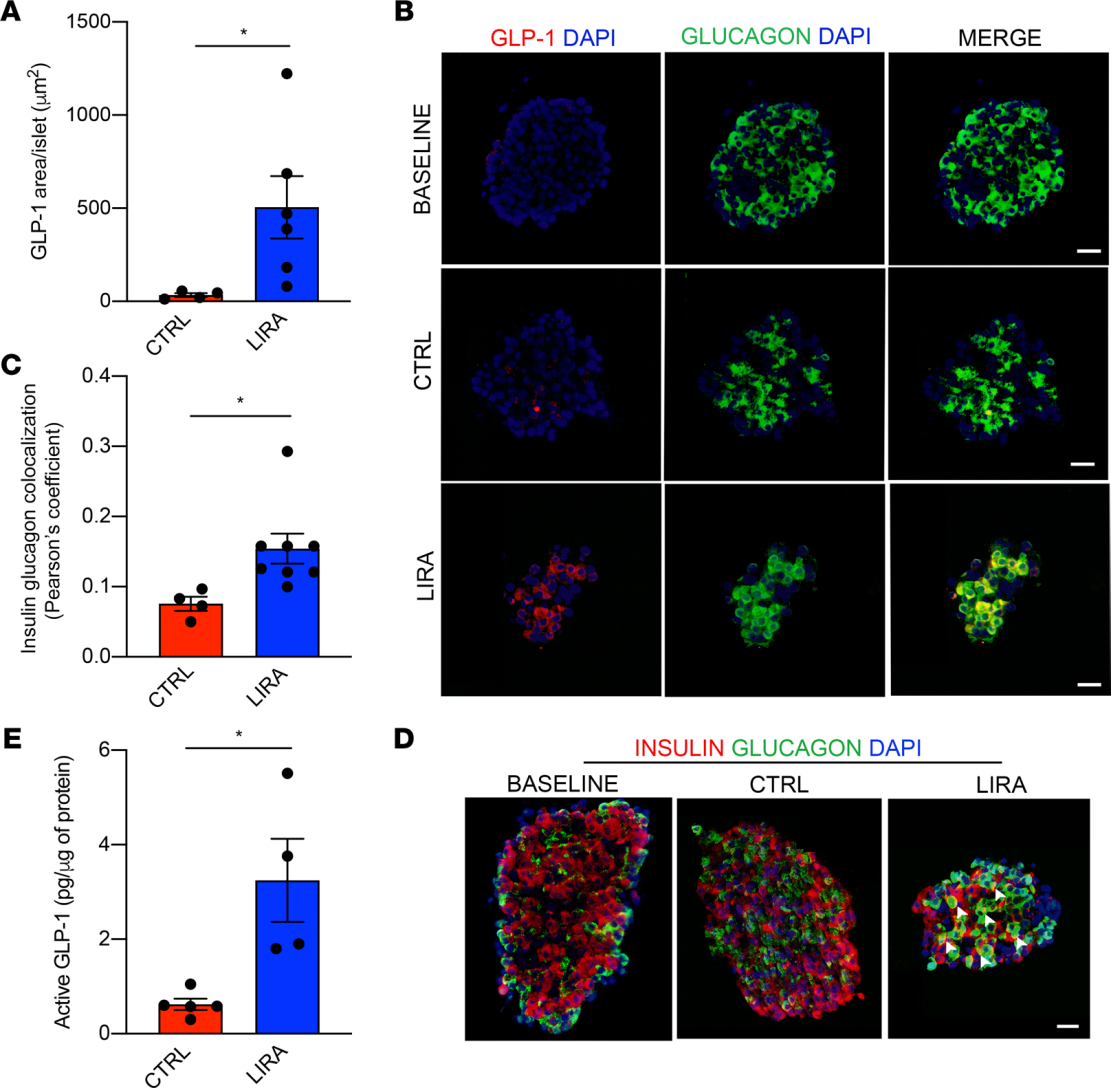
A GLP-1R agonist increases active GLP-1 production and expression and bihormonal insulin^+^ glucagon^+^ cells in human islets. (**A**) Average GLP-1 staining per islet. (**B**) Representative images of human islets at baseline and after 24 hours of saline or liraglutide treatment. Islets were immunostained for GLP-1 (red), glucagon (green), and DAPI. (**C**) Insulin and glucagon colocalization in the islet. (**D**) Islets immunostained for insulin (red), glucagon (green), and DAPI. Examples of bihormonal insulin^+^ glucagon^+^ cells are indicated by white arrows. (**E**) Active GLP-1 concentrations measured in lysate of human islets following 24 hours of saline or liraglutide treatment. Scale bar: 20 μm. *n* = 4–8 per group. *P < 0.05 by Student’s *t* test. See also Supplemental Figure 5.

To validate our findings that liraglutide increases *INS* and *PCSK1* expression in human α cells, immunostaining was performed on islets from a human donor (Supplemental Figure 5A) for GLP-1, insulin, and glucagon after 24 hours of treatment with saline or liraglutide. To control for the potential impact of stress induced by islet isolation and shipment, we performed immunostaining on islets that were fixed at the time of receipt (baseline). GLP-1^+^ and bihormonal insulin^+^ glucagon^+^ cells were rarely detected in baseline islets and islets treated with saline (Figure 5, A–D). Liraglutide treatment increased GLP-1^+^ and bihormonal insulin^+^ glucagon^+^ cells (Figure 5, A–D; *P* < 0.05). These data demonstrate that the effect of liraglutide treatment to increase *PCSK1* mRNA expression in a subcluster of α cells is associated with the expected increase in islet GLP-1 expression. Furthermore, these data show that the effect of liraglutide to increase coexpression of *INS* and *GCG* mRNA is reflected at the level of protein.

To confirm that increases in islet GLP-1 expression correspond with increases in the active form of GLP-1, we measured active GLP-1 in islet protein lysates from islets from a human donor (Supplemental Figure 5B) following 24 hours of treatment with saline or liraglutide. Human islets treated with liraglutide produced increased active GLP-1 as compared with controls (Figure 5E; *P* < 0.05). These results confirm previous work demonstrating that α cells can produce active GLP-1 (30, 67) and, further, that active GLP-1 production is amplified in response to a GLP-1R agonist. These findings are also consistent with previous work outlining the posttranslational processing of proglucagon by PC1/3. More specifically, when proglucagon is sorted into immature secretory granules, proglucagon is cleaved into glicentin and major proglucagon fragment (MPGF) by PC2 and/or PC1/3, respectively (68–70). Within mature secretory granules, once MPGF is generated, PC1/3 will first preferentially cleave MPGF at R77 and then cleave between R109 and R110, which renders the active forms of GLP-1, GLP-1(7–37) and GLP-1(7–36) (68, 71) as well as the N-terminally extended form of GLP-1 (68–72).

### β Cell GLP-1R signaling increases bihormonal insulin^+^ glucagon^+^ islet cells.

**Figure 6:**
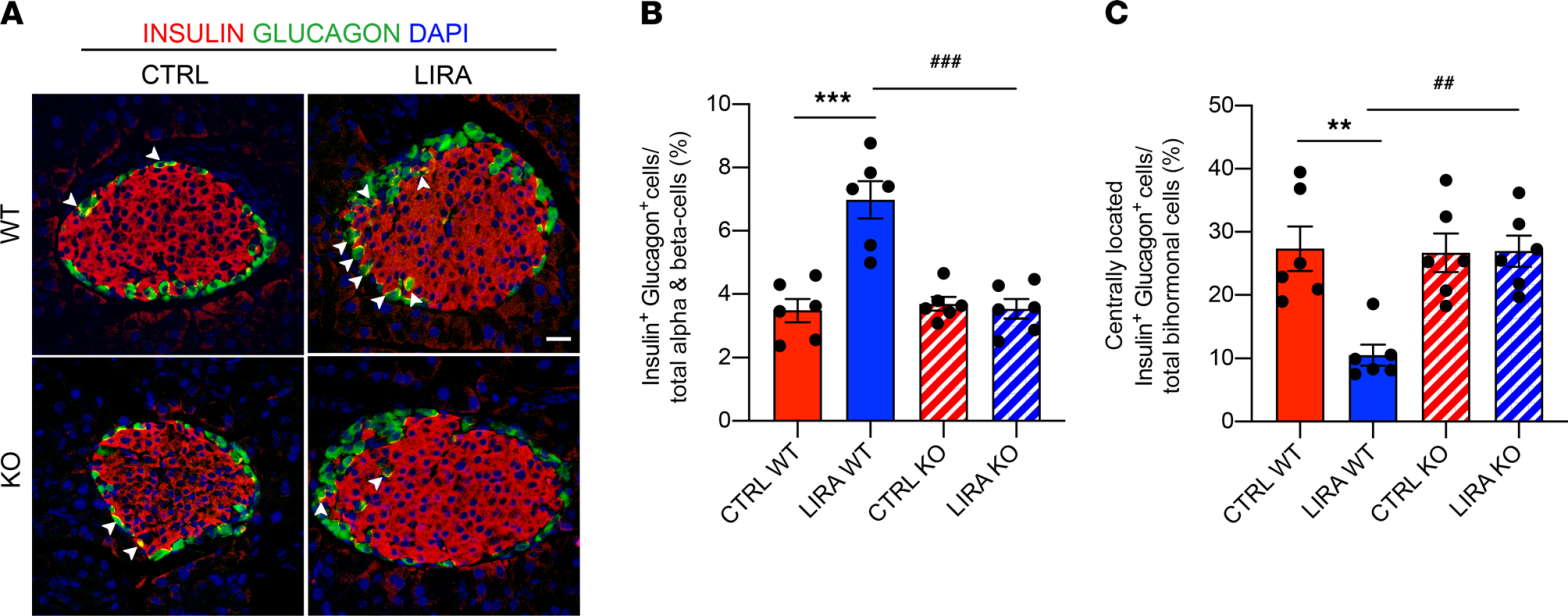
β Cell GLP-1R signaling increases bihormonal insulin^+^ glucagon^+^ islet cells in mice. (**A**) Representative images of pancreas sections immunostained for insulin (red), glucagon (green), and DAPI in β cell GLP-1R WT and KO mice treated with saline (CTRL) or liraglutide (LIRA). Examples of bihormonal insulin^+^ glucagon^+^ cells indicated by white arrows. (**B**) Percentage of bihormonal insulin^+^ glucagon^+^ cells per islet. (**C**) Percentage of centrally located bihormonal insulin^+^ glucagon^+^ cells per islet. Data are presented as mean ± SEM. *n* = 6 per group. **P < 0.01, ***P < 0.001 CTRL WT vs. LIRA WT, ^##^*P* < 0.01, ^###^*P <* 0.001 LIRA WT vs. LIRA KO by 2-factor ANOVA. Scale bar: 20 μm.

Previous reports found that the GLP-1R is robustly expressed in β cells, with some expression in δ cells, but is not found in α cells (73–75). However, other studies report that a small subpopulation (approximately 5%–10%) of α cells express the GLP-1R (76, 77). To determine if the observed effects of liraglutide to increase bihormonal insulin^+^ glucagon^+^ cells are mediated by the β cell GLP-1R, we assessed the effect of liraglutide treatment on insulin and glucagon coexpression in our β cell GLP-1R–KO mouse line. Mice were studied as described in Figure 1A, and pancreatic sections were costained for insulin and glucagon. Liraglutide treatment increased the percentage of bihormonal insulin^+^ glucagon^+^ cells relative to the total number of insulin- and glucagon-expressing cells per islet in WT mice but not in KO mice (Figure 6, A and B; *P* < 0.01). Furthermore, the percentage of bihormonal insulin^+^ glucagon^+^ cells was higher in liraglutide-treated WT mice compared with that in KO mice (Figure 6, A and B; *P* < 0.001). To determine the nature of the increase in bihormonal islet cells, we quantified the proportion of bihormonal insulin^+^ glucagon^+^ cells that were centrally located. The percentage of bihormonal insulin^+^ glucagon^+^ cells located centrally within the islet was lower in liraglutide-treated WT mice compared with that in saline-treated WT mice and liraglutide-treated KO mice (Figure 6C; *P* < 0.001). Together, these data demonstrate that liraglutide treatment increases bihormonal insulin^+^ glucagon^+^ cells at the islet periphery in a β cell GLP-1R–dependent fashion.

## Discussion

Herein, we report that pharmacologic stimulation of the β cell GLP-1R activates α cell PC1/3 and GLP-1 expression. Furthermore, we found that treatment of human islets with a GLP-1R agonist increases active GLP-1 expression and increases *PCSK1* expression in a subcluster of α cells. To generate this data set, we applied DART-Seq, a scRNA-Seq technique, to the study of human islets. Therefore, embedded in this manuscript is validation of a potentially novel scRNA-Seq approach to study human islets that offers higher resolution than other currently available platforms. In addition to increasing *PCSK1*, liraglutide treatment increases the relative abundance of other β cell–associated genes in the same subcluster of α cells. Finally, we found that the effect of liraglutide to increase bihormonal insulin^+^ glucagon^+^ cells is mediated by the β cell GLP-1R in mice. Together, our data demonstrate that our findings that β cell GLP-1R signaling increases α cell PC1/3 and GLP-1 expression in mice have translational relevance in human islets. Furthermore, these data reveal that the mechanism by which β cell GLP-1R signaling turns on α cell GLP-1 expression may be part of a broader pathway in which the α cell becomes an immature β-like cell. Thus, these data reveal that the β cell GLP-1R represents a pathway with which to target α cell GLP-1 and that this pathway is translationally relevant in human islets.

*PCSK1* is highly expressed in β cells, as it is required for proinsulin processing. Given that gene expression changes rarely happen in isolation, it is not surprising that liraglutide also increased the expression of other canonical β cell genes in α-1 cells. It is increasingly recognized that β cells and α cells exhibit significant plasticity. β Cells can be generated from α cells that first pass through a bihormonal insulin^+^ glucagon^+^ state (52, 78–84). Our DART-Seq data are consistent with those in previous work demonstrating that increased GLP-1R signaling promotes α cell conversion to β-like cells in mice (85, 86). Furthermore, α cell GLP-1 production is seen in immature pro-α cells (87–89). Moreover, we did not observe any liraglutide-mediated differential expression of human β cell dedifferentiation markers, such as *FGF1* and *FGF2*, MYC proto-oncogene (*MYC*)*,* SRY-box transcription factor 9 (*SOX9*), or Hes family BHLH transcription factor 1 (*HES1*) (90). Our IHC studies on human and mouse islets confirmed our DART-Seq findings that liraglutide increases bihormonal insulin^+^ glucagon^+^ cells. Notably, in mice, these bihormonal insulin^+^ glucagon^+^ cells were concentrated on the periphery, where α cells tend to reside. Nevertheless, whether β cell GLP-1R–induced α cell GLP-1 production is associated with a shift back to an immature developmental stage with β cell–like properties is unknown and will be a focus of future lineage tracing studies.

Our DART-Seq data also provide insight into the importance of islet endocrine cell heterogeneity under basal conditions and GLP-1R–stimulated conditions. While previous scRNA-Seq studies have focused primarily on β cell heterogeneity (32, 33), we found that both β cells and α cells exhibit pronounced heterogeneity that is driven, at least in part, by transcriptomic differences in cell maturity. In particular, we observed that the α-1 subcluster expressed low levels of *ARX*, *IRX2*, *CRYBA2*, *ALDH1A1*, and *TM4SF4*. Studies have shown that these genes are crucial markers of α cell fate and maturity (32, 50–52, 91). Based on their transcriptomic profile, it is tempting to hypothesize that α-1 cells are immature α cells that are pliable to a cell type conversion. This is supported by previous findings that ablation of *Arx* in neonatal α cells results in an α-to-β–like conversion through an intermediate bihormonal state, while ablation of *Arx* in adult mice does not (83).

Of note, our mouse studies were conducted in animals maintained on a HFD. Additionally, while the human islet donors did not have T2DM, these islet donors had BMIs consistent with an overweight condition. Obesity and T2DM decrease L cell–derived and increase α cell–derived GLP-1 in rodents and humans (30, 67, 92–99). Indeed, α cells exhibit a graded increase in GLP-1 synthesis with increasing metabolic stress (16, 30, 67, 92, 94, 100–103). Oral glucose tolerance and GSIS are impaired in HFD-fed β cell GLP-1R–KO mice compared with those in WT mice; however, this impairment is lost in low-fat diet–feeding conditions (9, 104). Previous work reports that the GLP-1R agonist, exendin-4, stimulates β cell proliferation in juvenile but not adult human islets (73). These data, in conjunction with our DART-Seq data generated in islets from older human donors, suggest that age and other metabolic stressors may drive α cell conversion to compensate for a loss of the β cell proliferative response to GLP-1. Therefore, it will be of interest to determine the effect of metabolic health on the effects of GLP-1R agonists in future work.

Although this study makes a critical contribution to the literature, there are limitations. In particular, our studies focused primarily on gene and protein expression and not on functional outcomes, such as secretion. Furthermore, our translational outcomes were based on isolated human islets. Future work is needed to determine the effects of this system on α cell and β cell secretory function.

In conclusion, these data reveal a previously unknown role for liraglutide in the regulation of α cell proglucagon processing and point to a potential effect on α cell fate. To this end, we applied a powerful scRNA-Seq tool to human islets, which will serve as a critical resource for the continued investigation of islet cell–type fate changes in the pathogenesis of T2DM. In addition to mechanistic and methodological insights, our findings offer hope for pharmaceutical interventions that can target α cell GLP-1 production to restore islet function and treat T2DM.

## Methods

### Animal studies.

Study mice were individually housed and maintained in a temperature and humidity-controlled room, with a 14/10-hour-light-dark cycle. Tamoxifen-inducible β cell–specific *Glp-1r*–KO (MIP-Cre-hGlp-1r) mice were generated in-house from our colony. This mouse line was originally developed by crossing humanized *Glp-1r*–knockin mice (105) with MIP-Cre/ERT mice (106), both of which are on a C57BL/6 background. At 2 months of age, male and female tamoxifen-inducible β cell–specific *Glp-1r^β^* *^cell+/+^* (WT) and *Glp-1r^β^* *^cell–/–^* (KO) littermates were placed on a HFD consisting of ground chow (5012 LabDiets) supplemented with 3.4% butter fat, 8.5% tallow, 13.1% soybean oil, 3.5% mineral mix, and 1% vitamin mix (Dyets) for 6 weeks. Mice were switched to a HFD with 400 mg/kg diet tamoxifen citrate (Envigo, TD.170935) for the duration of the study in order to induce GLP-1R knockdown. Two weeks after the start of HFD with tamoxifen, mice were given twice daily (08:00 and 16:00) injections of either saline or liraglutide (200 μg/kg) for 2 weeks. Mice were on HFD for a total of 8 weeks before intervention with liraglutide or saline. Body weights were matched between groups at the start of liraglutide/saline treatment. Following 2 weeks of liraglutide/saline treatment, mice were fasted overnight (12 hours) and given a last dose of liraglutide/saline at 08:00. Two hours after the final dose of liraglutide/saline, mice were euthanized by an overdose of pentobarbital (200 mg/kg i.p.), and tissues were weighed and collected.

### Human islets.

Human islets were obtained from the Integrated islet Distribution Program. Upon arrival, islets were resuspended in serum media (RPMI-1640 + 10% FBS + 5 mM glucose [for a final glucose concentration of 16.1 mM] + 1% antibiotic-antimycotic) and allowed to recuperate in the incubator set at 37°C and 5.0% CO_2_. After approximately 36 hours, the islets were transferred into serum-free media (RPMI-1640 + 5 mM glucose) for 2 hours. After 2 hours, islets were subjected to either liraglutide (100 nM) (107, 108) or saline treatment for 12 hours (DART-Seq) (8, 85, 109) in order to assess changes in RNA expression or 24 hours (7, 85, 107) (IHC, active GLP-1 measurements) in order to assess changes in protein content, as previously validated (85). Following this 12-hour treatment, islets used for DART-Seq were washed with PBS and dissociated into single-cell suspension using trypsin. Following dissociation, islets cells were washed 3 times with PBS containing 0.01% BSA, followed by centrifugation at 352*g* for 5 minutes, and then resuspended in PBS containing 0.01% BSA at a concentration of approximately 250,000 cells/mL, which was used for downstream DROP-Seq and DART-Seq experiments.

### Immunohistochemistry (mouse pancreas).

Immunohistochemistry was performed, as previously described (9, 24, 110). In brief, pancreas samples were fixed in 4% paraformaldehyde and paraffin embedded. Sections were deparaffinized in a xylene ethanol series and placed in Tris-EDTA (TE) buffer for antigen retrieval (10 mM Tris, 1 mM EDTA, 0.05% Tween, pH = 9.0) and then blocked in 5% BSA. Sections were immunostained for insulin using a monoclonal anti-mouse antibody (Santa Cruz Biotechnology, SC-377071; 1:100) and for glucagon using a monoclonal anti-rabbit antibody (Santa Cruz Biotechnology, SC-7779R; 1:200). Separate sections were immunostained for GLP-1 using a monoclonal anti-mouse antibody (Abcam, ab26278; 1:100) and glucagon using a monoclonal anti-rabbit antibody (Santa Cruz Biotechnology, SC-7779R; 1:200). Separate sections were immunostained for PC1/3 using a monoclonal anti-mouse antibody (Abcam, ab3532; 1:200) and glucagon using a monoclonal anti-rabbit antibody (Santa Cruz Biotechnology, SC-514592; 1:200). Detection of primary antibodies was performed with Alexa Fluor 488 anti-rabbit (A11034), Alexa Flour 488 anti-mouse (A11001), Alexa Flour 633 anti-mouse (A21052), and Alexa Fluor 633 anti-rabbit (A21070) secondary antibodies (1:200) (Invitrogen). Nuclei were detected with DAPI (Invitrogen, P36962).

### Immunohistochemistry (human islets).

Human islets used for IHC were washed in PBS after 24 hours of liraglutide or saline treatment and then fixed in 10% neutral buffered formalin overnight at 4°C. Following fixation, islets were washed twice in cold PBS. After washing, the islets were collected at the bottom of a 1.5 mL tube with 1 mL liquid HistoGel (Epredia), which was subsequently placed in a tissue cassette and processed for paraffin embedding. The sample was dehydrated in increasing concentrations of ethanol for 30 minutes in each solution as follows: 50% ethanol, 75% ethanol, 90% ethanol, 95% ethanol, 100% ethanol (twice) and cleared in xylene (twice) before an overnight incubation in paraffin. After embedding in paraffin, 5 μM sections were cut for IHC. Samples were deparaffinized in xylene and rehydrated in serial ethanol dilutions. Antigen retrieval was performed for 20 minutes in boiling TE, pH 9. Blocking was performed with 5% BSA. Sections were immunostained for insulin using a monoclonal anti-mouse antibody (Santa Cruz Biotechnology, SC-377071; 1:100) and for glucagon using a monoclonal anti-rabbit antibody (Santa Cruz Biotechnology, SC-7779R; 1:200). Separate sections were immunostained for GLP-1 using a monoclonal anti-mouse antibody (Abcam, ab26278; 1:100) and glucagon using a monoclonal anti-rabbit antibody (Santa Cruz Biotechnology, SC-7779R; 1:200). Detection of primary antibodies was performed with Alexa Flour 488 anti-rabbit (A11034) and Alexa Fluor 633 anti-mouse (A21052) secondary antibodies (1:200) (Invitrogen). Nuclei were detected with DAPI (Invitrogen, P36962).

### Active GLP-1 production.

Human islets were treated as described for the human islet IHC. Following 24 hours of saline or liraglutide treatment, islets were washed with culture media containing 16.1 mM glucose for 1 hour, after which islets were washed with PBS. Islet proteins were extracted using RIPA buffer (10 mM Tris-HCl, pH 7.4, 150 mM NaCl, 0.1% SDS, 1% Triton X-100, 1% sodium deoxycholate, 5 mM EDTA, 1 mM NaF, 1 mM sodium orthovanadate, protease inhibitors, and 125 μM DPP-IV inhibitor) and quantified with the bicinchoninic acid protein assay kit (Pierce Chemical). Active GLP-1 content in human islet protein lysates was measured by sandwich electrochemiluminescence immunoassay (catalog K1503OD; Meso Scale Discovery). Active GLP-1 values were normalized for the total protein content extracted from the same islets.

### Image analysis.

Mouse pancreas image quantification and analysis were performed on all islets in a single longitudinal cross-section of the pancreas cut at 5 μM through approximately 1000 μM of the whole pancreas tissue depth, as previously validated (111, 112) (1 cross-section per mouse, per antibody combination; 43 islets per mouse on average). Human islet image quantification and analysis was performed on 5 μM slices of individual islets sectioned through the center of the islet (6 islets per condition on average). Islet imaging and quantification were performed in a blinded fashion. Islets were imaged using a Nikon Eclipse E400 fluorescent microscope with Olympus DP73 color camera. Islets were then manually traced and quantified using ImageJ software (NIH). Islets with centrally located α cells were identified by visually identifying glucagon^+^ cells located centrally to the 3 most peripheral cell layers, as previously described (24). The JACoP plugin for ImageJ (NIH) was used to calculate the colocalization rate for PC1/3 and glucagon for mouse islets and the colocalization rate for insulin and glucagon for human islets, as previously described (113). Colocalization of PC1/3 and glucagon, and insulin and glucagon was evaluated using Pearson’s correlation coefficient. Bihormonal insulin^+^ glucagon^+^ cells in mouse islets were expressed as the percentage of cells coexpressing insulin and glucagon relative to the total number of α and β cells, as previously described (114). Islets with centrally located bihormonal insulin^+^ glucagon^+^ cells were identified by visually identifying insulin^+^ glucagon^+^ cells located centrally to the 3 most peripheral cell layers.

### DART-Seq bead synthesis.

DART-Seq beads were prepared as previously described (23). Single-stranded DNA probe sequences were designed to complement regions of human *PCSK1*, *PCSK2*, *GCG*, *INS*, *ATF6*, *ATF4*, and *XBP1* genes. The probes were annealed to the complementary toehold sequences that also carry a 10 to 12 bp overhang of A-repeats (Supplemental Table 1). All oligos were resuspended in TE buffer at a concentration of 500 μM. Double-stranded toehold adapters were created by heating equal volumes (20 μL) of the probe and splint oligos in the presence of 50 mM NaCl. The reaction mixture was heated to 95°C and cooled to 14°C at a slow rate (–0.1°C/s). The annealed mixture of toehold probes was diluted with TE buffer to obtain a final concentration of 100 μM. Equal amounts of toehold probes were mixed, and the final mixture was diluted to obtain the desired probe concentration (10 pmoles). 16 μL of this pooled probe mixture was combined with 40 μL PEG-4000 (50% w/v), 40 μL T4 DNA ligase buffer, 72 μL water, and 2 μL T4 DNA Ligase (30 U/μL, Thermo Fisher). Roughly 12,000 beads were combined with the above ligation mix and incubated for 1 hour at 37°C in an Eppendorf ThermoMixer (15 second alternative mixing at 1800 rpm). After ligation, enzyme activity was inhibited (65°C for 3 minutes), and beads were quenched in ice water. To obtain the desired quantity of DART-Seq primer beads, 6–10 bead ligation reactions were performed in parallel. All reactions were pooled, and beads were washed once with 250 μL TE-SDS buffer and twice with TE-Tween 20 (TE-TW) buffer. DART-Seq primer beads were stored in TE-TW at 4°C.

### Single-cell library preparation.

Single-cell library preparation was carried out as described previously (35). Briefly, single cells were encapsulated with beads in a droplet using a microfluidics device (Drop-seq microfluidic chips, FlowJEM). After cell lysis, cDNA synthesis was carried out (Maxima Reverse Transcriptase, Thermo Fisher), followed by PCR (2× Kapa Hotstart Ready mix, VWR, 15 cycles). cDNA libraries were tagmented and PCR amplified (NextEra tagmentation kit, Illumina). Finally, libraries were pooled and sequenced (Illumina Nextseq 500, 20 × 55 bp).

### Single-cell transcriptome profiling in human islet cells.

We used bioinformatic tools described in ref. 35 to process raw sequencing reads and obtain the gene cell count matrices. These matrices were then processed using the Seurat package for downstream analysis (36). Individual donor data sets were filtered using the following quality control metrics: we excluded genes detected in fewer than 5 cells (per subject), we excluded cells with fewer than 500 UMIs, and we excluded cells with higher than 5% of mitochondrial genes. To account for bimodal expression of insulin and glucagon hormones in the same cell, approximate counts per million thresholds for each marker gene were used before classifying cell types. The threshold was created by calculating the differences between *INS* and *GCG* counts within each data matrix; the median value of the difference was used as the threshold.

Gene cell count matrices for control and liraglutide-treated samples were independently normalized with LogNormalize, and the top 2000 most variable genes were used to integrate the 2 data sets (37). Dimensionality reduction of the integrated data set was performed by principal component analysis (PCA). Unsupervised graph-based clustering was performed on the first 20 principal components. Data annotated with corresponding clusters were visualized by UMAP. Differential gene expression analysis was performed using Seurat’s FindMarkers function, which performs differential expression based on the nonparametric Wilcoxon’s rank-sum test. Sequencing data are available in the GEO repository (GSE163744).

### Pseudotime trajectory construction.

The α and β cell subclusters generated by Seurat were imported into *Monocle* (53) to construct single-cell pseudotime ordering using the default setting.

### GO term analysis.

GO term analysis was performed using the PANTHER classification system (version 14) using differentially expressed genes from α-1 as input and α-4 as reference list.

### Statistics.

Data are presented as mean ± SEM unless otherwise stated. For our mouse data, we did not detect a difference between sexes, so data for males and females are presented together in the figures. We have included all data separated by sex in Supplemental Figure 6. For our mouse data, all statistical analyses were performed using GraphPad Prism 8.00 for Mac. Data were analyzed by 2-way ANOVA with Bonferroni’s post test or 2-tailed Student’s *t* test, as indicated. Differences were considered significant at *P* < 0.05.

### Study approval.

All experiments were performed in accordance with the *Guide for the Care and Use of Laboratory Animals* (National Academies Press, 2011) and approved by the Institutional Animal Care and Use Committee of Cornell University. The use of human islets was exempt, as cadaveric specimens without unique identifying information were used.

## Author contributions

MS contributed to study design, collected and analyzed data, and wrote the manuscript. MMH contributed to study design, collected and analyzed data, and revised the manuscript. LRD, ISL, QCZ, JLW, JET, DJP, and DG collected and analyzed data and revised the manuscript. RC, KWS, AGO, and CGD contributed to study design and data interpretation and revised the manuscript. BPC supervised the study, contributed to study design and data analysis, and finalized the manuscript.

## Supplementary Material

Supplemental data

Supplemental Table 2

## Version 1 02/08/2021

Electronic publication

## References

[1] Gerich JE (1975). Abnormal pancreatic glucagon secretion and postprandial hyperglycemia in diabetes mellitus. JAMA.

[2] Henkel E (2005). Impact of glucagon response on postprandial hyperglycemia in men with impaired glucose tolerance and type 2 diabetes mellitus. Metabolism.

[3] Unger RH, Cherrington AD (2012). Glucagonocentric restructuring of diabetes: a pathophysiologic and therapeutic makeover. J Clin Invest.

[4] Capozzi ME (2019). β Cell tone is defined by proglucagon peptides through cAMP signaling. JCI Insight.

[5] Svendsen B (2018). Insulin secretion depends on intra-islet glucagon signaling. Cell Rep.

[6] Zhu L (2019). Intra-islet glucagon signaling is critical for maintaining glucose homeostasis. JCI Insight.

[7] Toso C (2010). Liraglutide, a long-acting human glucagon-like peptide 1 analogue, improves human islet survival in culture. Transpl Int.

[8] Langlois A (2016). Improvement of islet graft function using liraglutide is correlated with its anti-inflammatory properties. Br J Pharmacol.

[9] Garibay D (2016). β-cell glucagon-like peptide-1 receptor contributes to improved glucose tolerance after vertical sleeve gastrectomy. Endocrinology.

[10] Sandoval DA, D’Alessio DA (2015). Physiology of proglucagon peptides: role of glucagon and GLP-1 in health and disease. Physiol Rev.

[11] Baggio LL, Drucker DJ (2007). Biology of incretins: GLP-1 and GIP. Gastroenterology.

[12] Hjollund KR (2011). Dipeptidyl peptidase-4 inhibition increases portal concentrations of intact glucagon-like peptide-1 (GLP-1) to a greater extent than peripheral concentrations in anaesthetised pigs. Diabetologia.

[13] Mulvihill EE (2017). Cellular sites and mechanisms linking reduction of dipeptidyl peptidase-4 activity to control of incretin hormone action and glucose homeostasis. Cell Metab.

[14] Mulvihill EE (2018). Dipeptidyl peptidase inhibitor therapy in type 2 diabetes: control of the incretin axis and regulation of postprandial glucose and lipid metabolism. Peptides.

[15] Holst JJ, Deacon CF (2005). Glucagon-like peptide-1 mediates the therapeutic actions of DPP-IV inhibitors. Diabetologia.

[16] Kilimnik G (2010). Intraislet production of GLP-1 by activation of prohormone convertase 1/3 in pancreatic α-cells in mouse models of ß-cell regeneration. Islets.

[17] Chambers AP (2017). The role of pancreatic preproglucagon in glucose homeostasis in mice. Cell Metab.

[18] Hutch CR (2019). The role of GIP and pancreatic GLP-1 in the glucoregulatory effect of DPP-4 inhibition in mice. Diabetologia.

[19] Kim KS (2019). Glycemic effect of pancreatic preproglucagon in mouse sleeve gastrectomy. JCI Insight.

[20] Traub S (2017). Pancreatic α cell-derived glucagon-related peptides are required for β cell adaptation and glucose homeostasis. Cell Rep.

[21] Wideman RD (2007). A switch from prohormone convertase (PC)-2 to PC1/3 expression in transplanted alpha-cells is accompanied by differential processing of proglucagon and improved glucose homeostasis in mice. Diabetes.

[22] Wideman RD (2009). Transplantation of PC1/3-expressing alpha-cells improves glucose handling and cold tolerance in leptin-resistant mice. Mol Ther.

[23] Saikia M (2019). Simultaneous multiplexed amplicon sequencing and transcriptome profiling in single cells. Nat Methods.

[24] Garibay D (2018). β cell GLP-1R signaling alters α cell proglucagon processing after vertical sleeve gastrectomy in mice. Cell Rep.

[25] Jun LS (2014). A novel humanized GLP-1 receptor model enables both affinity purification and Cre-LoxP deletion of the receptor. PLoS One.

[26] Andrikopoulos S (2008). Evaluating the glucose tolerance test in mice. Am J Physiol Endocrinol Metab.

[27] Gupta D (2017). Temporal characterization of β cell-adaptive and -maladaptive mechanisms during chronic high-fat feeding in C57BL/6NTac mice. J Biol Chem.

[28] Paschen M (2019). Diet-induced β-cell insulin resistance results in reversible loss of functional β-cell mass. FASEB J.

[29] Campbell SA (2020). Human islets contain a subpopulation of glucagon-like peptide-1 secreting α cells that is increased in type 2 diabetes. Mol Metab.

[30] Marchetti P (2012). A local glucagon-like peptide 1 (GLP-1) system in human pancreatic islets. Diabetologia.

[31] Davis EM, Sandoval DA (2020). Glucagon-like peptide-1: actions and influence on pancreatic hormone function. Compr Physiol.

[32] Segerstolpe A (2016). Single-cell transcriptome profiling of human pancreatic islets in health and type 2 diabetes. Cell Metab.

[33] Fang Z (2019). Single-cell heterogeneity analysis and CRISPR screen identify key β-cell-specific disease genes. Cell Rep.

[34] Mawla AM, Huising MO (2019). Navigating the depths and avoiding the shallows of pancreatic islet cell transcriptomes. Diabetes.

[35] Macosko EZ (2015). Highly parallel genome-wide expression profiling of individual cells using nanoliter droplets. Cell.

[36] Satija R (2015). Spatial reconstruction of single-cell gene expression data. Nat Biotechnol.

[37] Stuart T (2019). Comprehensive integration of single-cell data. Cell.

[38] Camunas-Soler J (2020). Patch-seq links single-cell transcriptomes to human islet dysfunction in diabetes. Cell Metab.

[39] Muraro MJ (2016). A single-cell transcriptome atlas of the human pancreas. Cell Syst.

[40] Pipeleers D (1994). Physiologic relevance of heterogeneity in the pancreatic beta-cell population. Diabetologia.

[41] Kiekens R (1992). Differences in glucose recognition by individual rat pancreatic B cells are associated with intercellular differences in glucose-induced biosynthetic activity. J Clin Invest.

[42] Van Schravendijk CF (1992). Pancreatic beta cell heterogeneity in glucose-induced insulin secretion. J Biol Chem.

[43] Kameswaran V (2014). Epigenetic regulation of the DLK1-MEG3 microRNA cluster in human type 2 diabetic islets. Cell Metab.

[44] Kameswaran V (2018). The Dysregulation of the *DLK1*-*MEG3* locus in islets from patients with type 2 diabetes is mimicked by targeted epimutation of its promoter with TALE-DNMT constructs. Diabetes.

[45] You L (2016). Downregulation of long noncoding RNA Meg3 affects insulin synthesis and secretion in mouse pancreatic beta cells. J Cell Physiol.

[46] Mueckler M (1994). Facilitative glucose transporters. Eur J Biochem.

[47] Romer AI (2019). Murine perinatal β-cell proliferation and the differentiation of human stem cell-derived insulin-expressing cells require NEUROD1. Diabetes.

[48] Villasenor A (2010). Rgs16 and Rgs8 in embryonic endocrine pancreas and mouse models of diabetes. Dis Model Mech.

[49] Ruiz de Azua I (2010). RGS4 is a negative regulator of insulin release from pancreatic beta-cells in vitro and in vivo. Proc Nat Acad Sci U S A.

[50] Collombat P (2003). Opposing actions of Arx and Pax4 in endocrine pancreas development. Genes Dev.

[51] Dorrell C (2011). Transcriptomes of the major human pancreatic cell types. Diabetologia.

[52] Chakravarthy H (2017). Converting adult pancreatic islet α cells into β cells by targeting both Dnmt1 and Arx. Cell Metab.

[53] Trapnell C (2014). The dynamics and regulators of cell fate decisions are revealed by pseudotemporal ordering of single cells. Nat Biotechnol.

[54] Cigliola V (2016). Stress-induced adaptive islet cell identity changes. Diabetes Obes Metab.

[55] Azzarelli R (2018). Neurogenin3 phosphorylation controls reprogramming efficiency of pancreatic ductal organoids into endocrine cells. Sci Rep.

[56] Shimoda M (2011). The human glucagon-like peptide-1 analogue liraglutide preserves pancreatic beta cells via regulation of cell kinetics and suppression of oxidative and endoplasmic reticulum stress in a mouse model of diabetes. Diabetologia.

[57] Hetz C (2012). The unfolded protein response: controlling cell fate decisions under ER stress and beyond. Nat Rev Mol Cell Biol.

[58] Walter P, Ron D (2011). The unfolded protein response: from stress pathway to homeostatic regulation. Science.

[59] Xin Y (2018). Pseudotime ordering of single human β-cells reveals states of insulin production and unfolded protein response. Diabetes.

[60] Waeber G (1997). Insulin secretion is regulated by the glucose-dependent production of islet beta cell macrophage migration inhibitory factor. Proc Natl Acad Sci U S A.

[61] Koulmanda M (2008). Curative and beta cell regenerative effects of alpha1-antitrypsin treatment in autoimmune diabetic NOD mice. Proc Natl Acad Sci U S A.

[62] Sandstrom CS (2008). An association between Type 2 diabetes and alpha-antitrypsin deficiency. Diabet Med.

[63] Zhang B (2007). Alpha1-antitrypsin protects beta-cells from apoptosis. Diabetes.

[64] Terazono K (1990). Expression of reg protein in rat regenerating islets and its co-localization with insulin in the beta cell secretory granules. Diabetologia.

[65] Dean ED (2020). A primary role for α-cells as amino acid sensors. Diabetes.

[66] Menegaz D (2019). Mechanism and effects of pulsatile GABA secretion from cytosolic pools in the human beta cell. Nat Metab.

[67] Nie Y (2000). Regulation of pancreatic PC1 and PC2 associated with increased glucagon-like peptide 1 in diabetic rats. J Clin Invest.

[68] Dey A (2005). Significance of prohormone convertase 2, PC2, mediated initial cleavage at the proglucagon interdomain site, Lys70-Arg71, to generate glucagon. Endocrinology.

[69] Guizzetti L (2014). Two dipolar α-helices within hormone-encoding regions of proglucagon are sorting signals to the regulated secretory pathway. J Biol Chem.

[70] Rouillé Y (1994). Proglucagon is processed to glucagon by prohormone convertase PC2 in alpha TC1-6 cells. Proc Natl Acad Sci U S A.

[71] Rouillé Y (1997). Role of the prohormone convertase PC3 in the processing of proglucagon to glucagon-like peptide 1. J Biol Chem.

[72] Dhanvantari S (1996). Role of prohormone convertases in the tissue-specific processing of proglucagon. Mol Endocrinol.

[73] Dai C (2017). Age-dependent human β cell proliferation induced by glucagon-like peptide 1 and calcineurin signaling. J Clin Invest.

[74] DiGruccio MR (2016). Comprehensive alpha, beta and delta cell transcriptomes reveal that ghrelin selectively activates delta cells and promotes somatostatin release from pancreatic islets. Mol Metab.

[75] Tornehave D (2008). Expression of the GLP-1 receptor in mouse, rat, and human pancreas. J Histochem Cytochem.

[76] Ast J (2020). Super-resolution microscopy compatible fluorescent probes reveal endogenous glucagon-like peptide-1 receptor distribution and dynamics. Nat Commun.

[77] Richards P (2014). Identification and characterization of GLP-1 receptor-expressing cells using a new transgenic mouse model. Diabetes.

[78] Chung CH (2010). Pancreatic β-cell neogenesis by direct conversion from mature α-cells. Stem Cells.

[79] Collombat P (2009). The ectopic expression of Pax4 in the mouse pancreas converts progenitor cells into alpha and subsequently beta cells. Cell.

[80] Courtney M (2013). The inactivation of Arx in pancreatic α-cells triggers their neogenesis and conversion into functional β-like cells. PLoS Genet.

[81] Thorel F (2010). Conversion of adult pancreatic alpha-cells to beta-cells after extreme beta-cell loss. Nature.

[82] van der Meulen T, Huising MO (2015). Role of transcription factors in the transdifferentiation of pancreatic islet cells. J Mol Endocrinol.

[83] Wilcox CL (2013). Pancreatic α-cell specific deletion of mouse Arx leads to α-cell identity loss. PLoS One.

[84] Yang YP (2011). Context-specific α- to-β-cell reprogramming by forced Pdx1 expression. Genes Dev.

[85] Lee YS (2018). Glucagon-like peptide 1 increases β-cell regeneration by promoting α- to β-cell transdifferentiation. Diabetes.

[86] Zhang Z (2019). A new way for beta cell neogenesis: transdifferentiation from alpha cells induced by glucagon-like peptide 1. J Diabetes Res.

[87] Kim S (2020). Molecular and genetic regulation of pig pancreatic islet cell development. Development.

[88] Stanojevic V, Habener JF (2015). Evolving function and potential of pancreatic alpha cells. Best Pract Res Clin Endocrinol Metab.

[89] Wilson ME (2002). Expression pattern of IAPP and prohormone convertase 1/3 reveals a distinctive set of endocrine cells in the embryonic pancreas. Mech Dev.

[90] Diedisheim M (2018). Modeling human pancreatic beta cell dedifferentiation. Mol Metab.

[91] Furuyama K (2019). Diabetes relief in mice by glucose-sensing insulin-secreting human α-cells. Nature.

[92] Moffett RC (2014). Incretin receptor null mice reveal key role of GLP-1 but not GIP in pancreatic beta cell adaptation to pregnancy. PLoS One.

[93] Muscelli E (2008). Separate impact of obesity and glucose tolerance on the incretin effect in normal subjects and type 2 diabetic patients. Diabetes.

[94] O’Malley TJ (2014). Progressive change of intra-islet GLP-1 production during diabetes development. Diabetes Metab Res Rev.

[95] Rask E (2001). Impaired incretin response after a mixed meal is associated with insulin resistance in nondiabetic men. Diabetes Care.

[96] Richards P (2016). High fat diet impairs the function of glucagon-like peptide-1 producing L-cells. Peptides.

[97] Stumvoll M (2002). Clinical characterization of insulin secretion as the basis for genetic analyses. Diabetes.

[98] Toft-Nielsen MB (2001). Determinants of the impaired secretion of glucagon-like peptide-1 in type 2 diabetic patients. J Clin Endocrinol Metab.

[99] Vilsboll T (2001). Reduced postprandial concentrations of intact biologically active glucagon-like peptide 1 in type 2 diabetic patients. Diabetes.

[100] Dusaulcy R (2016). Functional and molecular adaptations of enteroendocrine L-cells in male obese mice are associated with preservation of pancreatic α-cell function and prevention of hyperglycemia. Endocrinology.

[101] Kumar DP (2016). Activation of transmembrane bile acid receptor TGR5 modulates pancreatic islet α cells to promote glucose homeostasis. J Biol Chem.

[102] Masur K (2005). Basal receptor activation by locally produced glucagon-like peptide-1 contributes to maintaining beta-cell function. Mol Endocrinol.

[103] Whalley NM (2011). Processing of proglucagon to GLP-1 in pancreatic α-cells: is this a paracrine mechanism enabling GLP-1 to act on β-cells?. J Endocrinol.

[104] Smith EP (2014). The role of β cell glucagon-like peptide-1 signaling in glucose regulation and response to diabetes drugs. Cell Metab.

[105] Jun LS (2014). A novel humanized GLP-1 receptor model enables both affinity purification and Cre-LoxP deletion of the receptor. PLoS One.

[106] Wicksteed B (2010). Conditional gene targeting in mouse pancreatic β-cells: analysis of ectopic Cre transgene expression in the brain. Diabetes.

[107] Wang L (2014). GLP-1 analog liraglutide enhances proinsulin processing in pancreatic β-cells via a PKA-dependent pathway. Endocrinology.

[108] Bregenholt S (2005). The long-acting glucagon-like peptide-1 analogue, liraglutide, inhibits beta-cell apoptosis in vitro. Biochem Biophys Res Commun.

[109] Langlois A (2016). In vitro and in vivo investigation of the angiogenic effects of liraglutide during islet transplantation. PLoS One.

[110] McGavigan AK (2017). TGR5 contributes to glucoregulatory improvements after vertical sleeve gastrectomy in mice. Gut.

[111] Golson ML (2014). Automated quantification of pancreatic β-cell mass. Am J Physiol Endocrinol Metab.

[112] Steil GM (2001). Adaptation of beta-cell mass to substrate oversupply: enhanced function with normal gene expression. Am J Physiol Endocrinol Metab.

[113] Bolte S, Cordelieres FP (2006). A guided tour into subcellular colocalization analysis in light microscopy. J Microsc.

[114] Riedel MJ (2012). Immunohistochemical characterisation of cells co-producing insulin and glucagon in the developing human pancreas. Diabetologia.

