# Supplementary material for: GLP-1 receptor signaling increases PCSK1 and β cell features in human α cells
Source: JCI Insight. 2021 Feb 8;6(3):e141851. doi: 10.1172/jci.insight.141851 (PMC7934853; doi:10.1172/jci.insight.141851)
Supplement: Supplemental data [file jciinsight-6-141851-s209.pdf]

SUPPLEMENTAL FIGURE LEGENDS

Supplemental Figure 1. (**A**) Body weight, (**B**) cumulative food intake from day 5-14 of liraglutide or saline treatment, (**C**) subcutaneous (SC), mesenteric (MES), perigonadal (PG) and retroperitoneal (RP) adipose depot weights, (**D**) total white adipose tissue (WAT) weight, (**E**) brown adipose tissue (BAT) weight, and (**F**) fasting blood glucose in saline (CTRL) or liraglutide- (LIRA) treated WT and KO mice. Data are represented as mean ± SEM. *n* = 6 per group. **P*<0.05 CTRL WT vs LIRA WT by Student's t-test. $^{\$}$*P*<0.05 CTRL KO vs LIRA KO by one-tailed Student's t-test.

Supplemental Figure 2. (**A**) UMAP of human islet cells that were subjected to either DROP-seq or DART-seq. (**B**) Donor information of the human islet sample used for the study. *n* = 1.

Supplemental Figure 3. (**A**) Table of islet donor information. (**B**) Cell marker expression overlaid on the UMAP of human islets treated with CTRL or LIRA. (**C**) UMAP of human islets treated with either saline (CTRL) or liraglutide (LIRA). *n* = 3.

Supplemental Figure 4. (**A**) Table showing average log-fold change (avg_logFC) in the expression of *INS*, *IAPP*, *PCSK1* and *MAFA* upon liraglutide treatment, within the alpha-cell sub-clusters. n.s. = not significant. (**B**) Volcano plot showing liraglutide-induced differential gene expression in delta-cells. *n* = 3.

Supplemental Figure 5. (**A**) Table of islet donor information for human islets used for IHC outcomes. (**B**) Table of islet donor information for human islets used for active GLP-1 measures.

Supplemental Figure 6. Comparison of males vs females. (**A**) Body weight, (**B**) cumulative food intake from day 5-14 of liraglutide or saline treatment, (**C**) total white adipose tissue (WAT) weight,

(**D**) brown adipose tissue (BAT) weight, (**E**) subcutaneous (SC), mesenteric (MES), perigonadal (PG) and retroperitoneal (RP) adipose depot weights, (**F**) fasting blood glucose in saline (CTRL) or liraglutide- (LIRA) treated WT and KO mice. (**G**) Average GLP-1 staining per islet. (**H**) PC1/3 co-localization with glucagon. (**I**) Percentage of islets with centrally located alpha-cells. (**J**) Percentage of bi-hormonal insulin$^+$ glucagon$^+$ positive cells per islet. (**K**) Percentage of centrally located bi-hormonal insulin$^+$ glucagon$^+$ positive cells per islet. Data are presented as mean ± SEM. n = 6 per group (3 males, 3 females). $^*P<0.05$ CTRL WT vs LIRA WT, $^+P<0.05$ CTRL KO vs LIRA KO, $^\#P<0.05$ LIRA WT vs LIRA KO by two-factor ANOVA; $^\$P<0.05$ CTRL WT vs LIRA WT, $^+P<0.05$ CTRL WT vs CTRL KO by two-tailed Student's t-test.

SUPPLEMENTAL TABLES

Supplemental Table 1. Sequence information of DNA primers used as the probes and toeholds for DART-seq beads. Related to Figure 2.

Name	Sequence (5'-3')
**PCSK1_Probe**	/5Phos/GCC GGT CGT CTC TGT GCT TG
**PCSK1_Toehold**	GAG ACG ACC GGC AAA AAA AAA AAA
**GCG_Probe**	/5Phos/TTG GTC TGA ATC AAC CAG TT
**GCG_Toehold**	GAT TCA GAC CAA AAA AAA AAA AAA
**PCSK2_Probe**	/5Phos/CTG GTC GAT GTA CGG GGC AC
**PCSK2_Toehold**	TAC ATC GAC CAG AAA AAA AAA AAA
**PCSK1n_Probe**	/5Phos/AGG CGT TTC ACA CGC AGC AG
**PCSK1n_Toehold**	TGT GAA ACG CCT AAA AAA AAA AAA
**XBP1U_Probe**	/5Phos/TCC AGG CTG GCA GGC TCT GG
**XBP1U_Toehold**	TGC CAG CCT GGA AAA AAA AAA AAA
**XBP1s_Probe**	/5Phos/TGG GTC CAA GTT GTC CAG AA
**XBP1s_Toehold**	AAC TTG GAC CCA AAA AAA AAA AAA
**ATF6_Probe**	/5Phos/CCA CAG AGG CAA CCC ACG TT
**ATF6_Toehold**	TTG CCT CTG TGG AAA AAA AAA AAA
**ATF4_Probe**	/5Phos/CAA GAC AGC AGC CAC TAG GT
**ATF4_Toehold**	GCT GCT GTC TTG AAA AAA AAA AAA

Supplemental Table 2. List of top 100 genes that are differentially expressed in the liraglutide-treated versus control condition for every alpha and beta sub-cluster. Related to Figure 4.

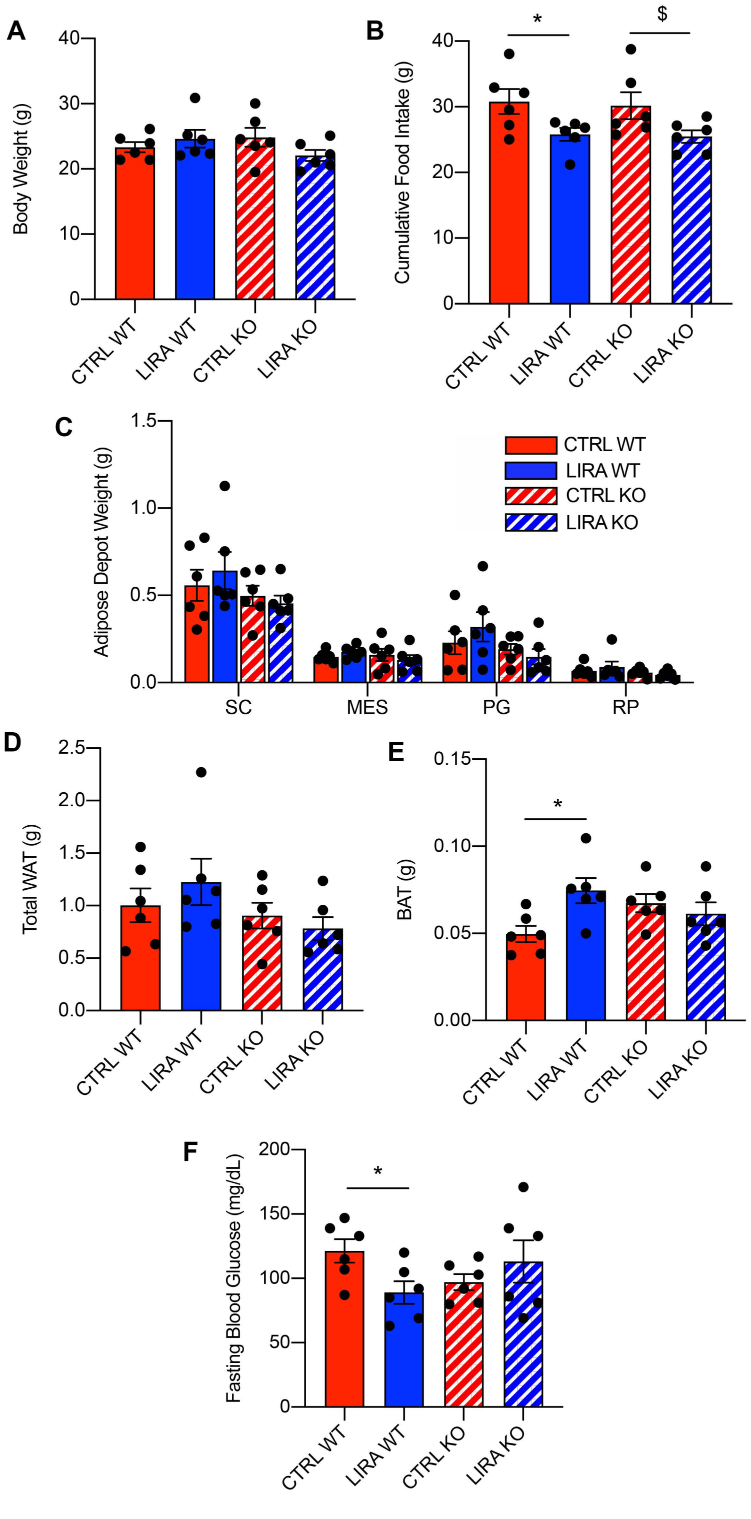
A
Body Weight (g)
0
10
20
30
40
CTRL WT
LIRA WT
CTRL KO
LIRA KO
B
Cumulative Food Intake (g)
*
$
C
Adipose Depot Weight (g)
0.0
0.5
1.0
1.5
CTRL WT
LIRA WT
CTRL KO
LIRA KO
SC
MES
PG
RP
D
Total WAT (g)
0.0
0.5
1.0
1.5
2.0
2.5
E
BAT (g)
0.00
0.05
0.10
0.15
*
F
Fasting Blood Glucose (mg/dL)
0
50
100
150
200
*

A

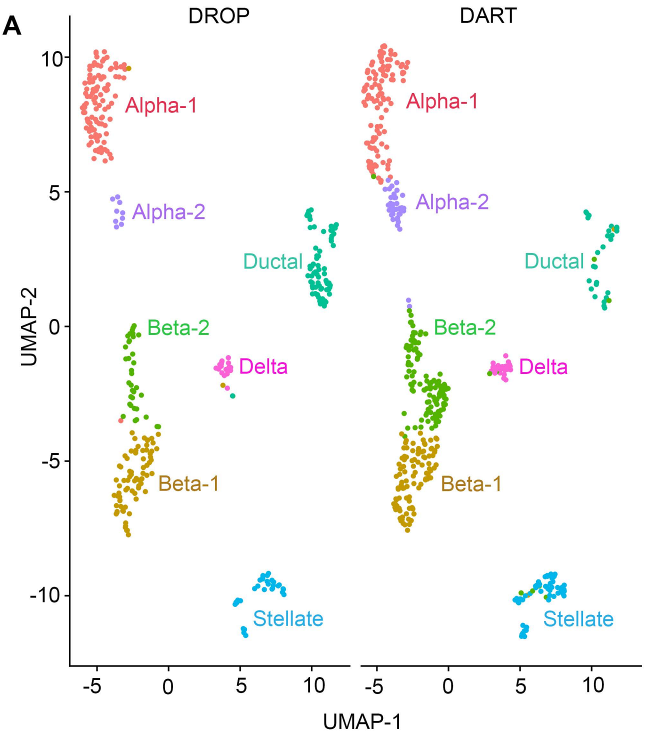

B

Age	Sex	BMI	HbA1c (%)
40	Male	30.7	5.2

A

Donor	Age	Sex	BMI	HbA1c (%)
D1	61	Male	28.8	5.1
D2	66	Male	27.0	4.7
D3	40	Male	30.7	5.2

B

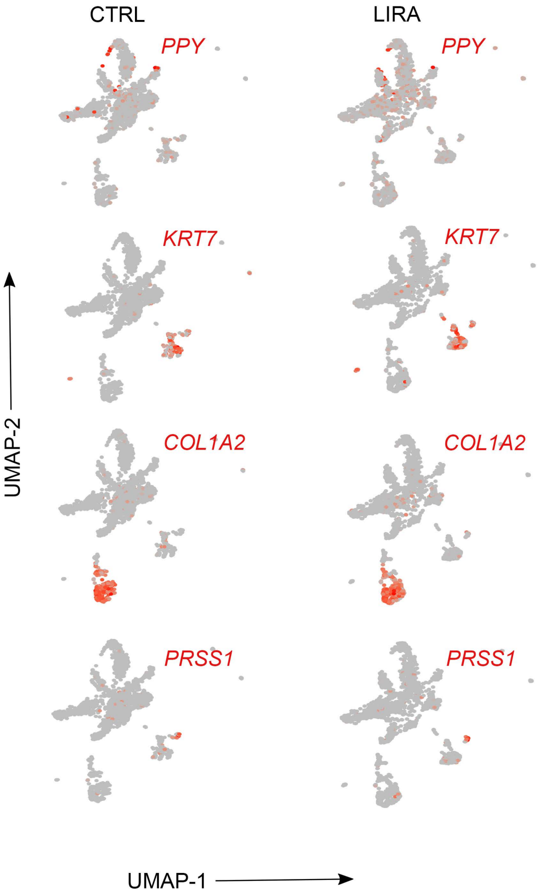

C

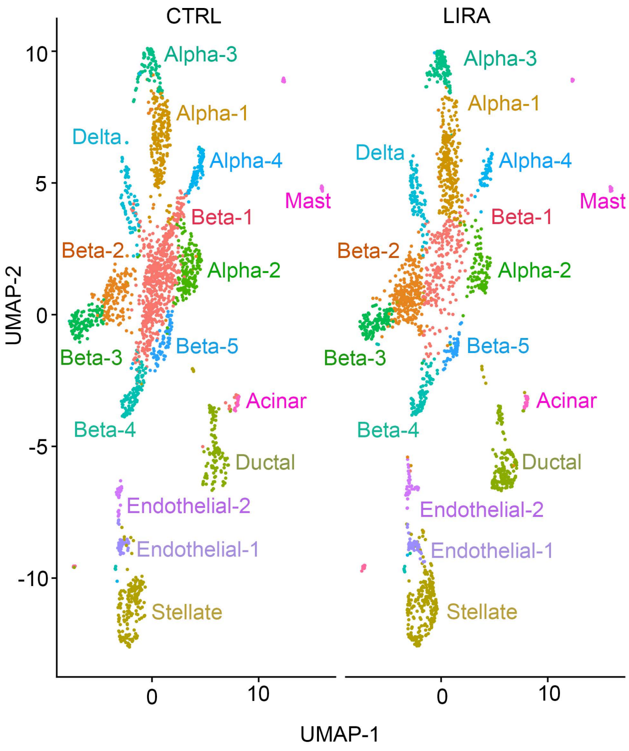

A

GENE	Alpha-1		Alpha-2		Alpha-3		Alpha-4	
	p-value	avg_logFC	p-value	avg_logFC	p-value	avg_logFC	p-value	avg_logFC
INS	9.21E-55	1.38824	1.14E-07	-0.561265	n.s.	n.s.	8.19E-08	-0.394865
IAPP	2.65E-51	1.66715	n.s.	n.s.	1.08E-12	1.616480	n.s.	n.s.
PCSK1	5.04E-46	1.63618	n.s.	n.s.	0.0111255	1.794607	n.s.	n.s.
MAFA	0.0005178	0.4388932	n.s.	n.s.	n.s.	n.s.	n.s.	n.s.

B

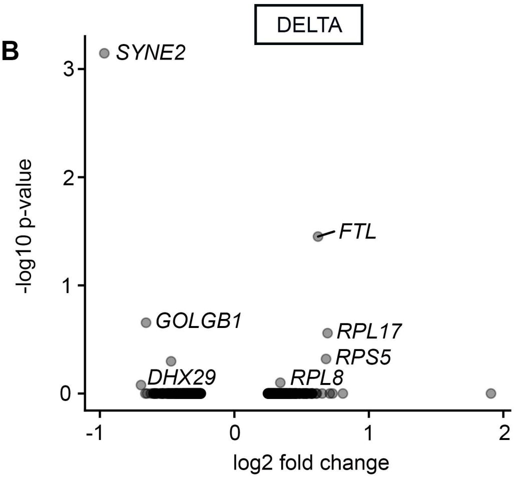

A

Age	Sex	BMI	HbA1c (%)
37	Male	31.9	5.6

B

Age	Sex	BMI	HbA1c (%)
25	Male	31.3	5.9

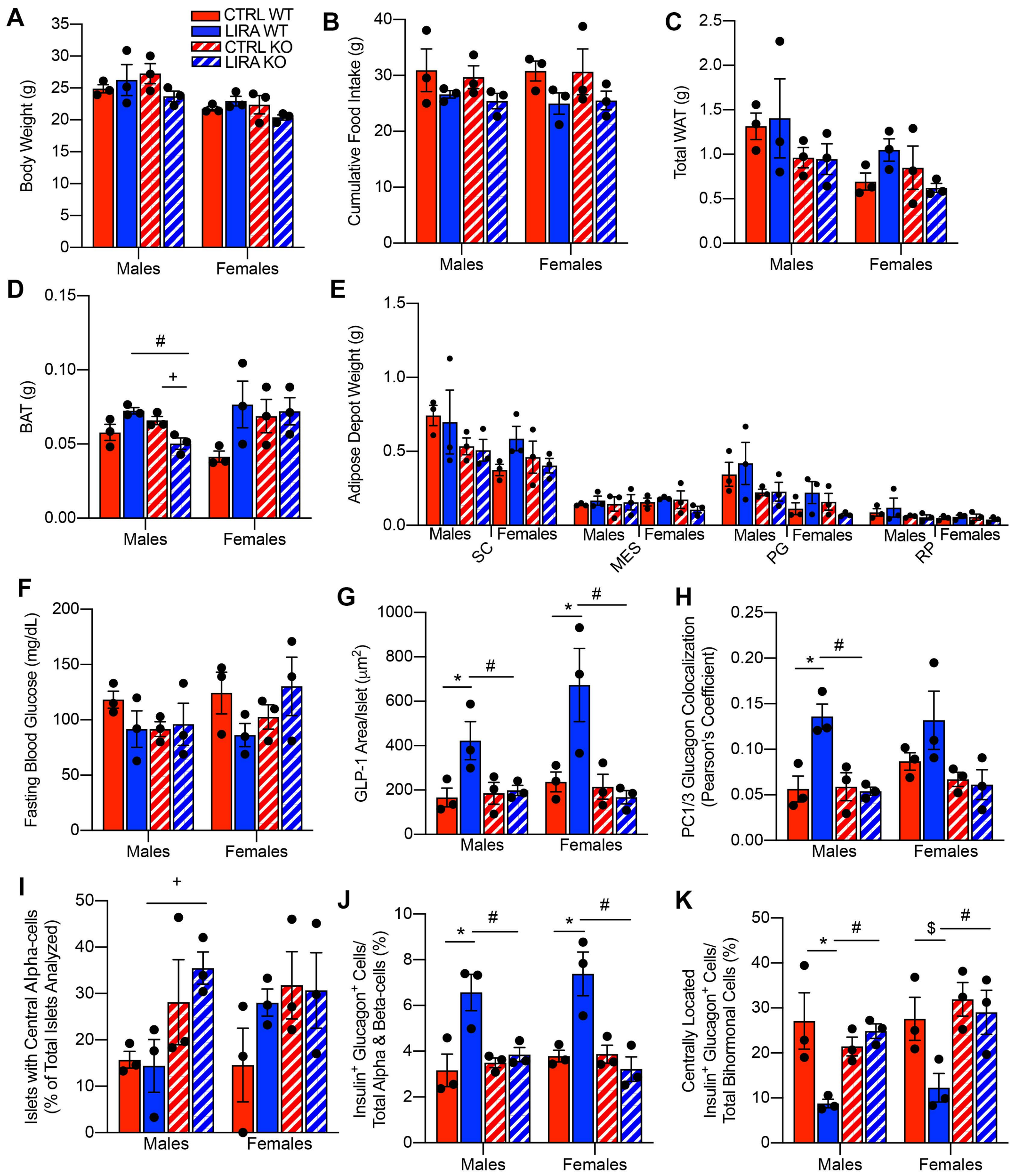

A
B
C
D
E
F
G
H
I
J
K
CTRL WT
LIRA WT
CTRL KO
LIRA KO
Body Weight (g)
Cumulative Food Intake (g)
Total WAT (g)
BAT (g)
Adipose Depot Weight (g)
SC
MES
PG
RP
Fasting Blood Glucose (mg/dL)
GLP-1 Area/Islet (μm²)
PC1/3 Glucagon Colocalization (Pearson's Coefficient)
Islets with Central Alpha-cells (% of Total Islets Analyzed)
Insulin⁺ Glucagon⁺ Cells/ Total Alpha & Beta-cells (%)
Centrally Located Insulin⁺ Glucagon⁺ Cells/ Total Bihormonal Cells (%)
Males
Females
